# Tregs With High CD29 Expression Promote Cell Adhesion and Contribute to the Malignant Transformation of MASLD

**DOI:** 10.1111/liv.70421

**Published:** 2025-11-07

**Authors:** Yuming Lu, Luyin Liu, Mengya Zhou, Minghui Zou, Linling Ju, Dengfu Yao, Min Yao

**Affiliations:** ^1^ Department of Immunology Medical School of Nantong University Nantong China; ^2^ Department of Pathology The Second Affiliated Hospital of Soochow University Suzhou China; ^3^ Research Center of Clinical Medicine Affiliated Hospital of Nantong University Nantong China

**Keywords:** CD29, cell adhesion, lipid accumulation, metabolic fatty liver disease, Tregs

## Abstract

**Background and Aim:**

Regulatory T cells (Tregs) are highly enriched in the metabolic dysfunction‐associated steatotic liver disease (MASLD) microenvironment, but their role in driving metabolic dysfunction‐associated steatohepatitis (MASH) progression to hepatocellular carcinoma (HCC) remains unclear. Here, it is demonstrated that integrin β1 (ITGβ1, CD29) expression is upregulated by Tregs, enhancing cell adhesion and driving the malignant transformation of MASLD.

**Methods:**

A MASLD mouse model was established via high‐fat diet (HFD) and 2‐acetylamino fluorene (2‐AAF). Single‐cell RNA sequencing (scRNA‐seq) and flow cytometry were employed to quantitatively analyse the distribution of T lymphocytes and their subsets in the livers of MASLD mice, with particular focus on the Treg subset. Subsequently, a Treg subset with high CD29 expression was identified through unsupervised clustering analysis. Further validation using single‐cell sequencing data and in vitro functional assays demonstrated that Tregs promote cell adhesion by upregulating CD29, thereby driving the progression of MASH to HCC. Finally, the effect of CD29 knockdown on the Tregs‐induced malignant transformation of MASLD was studied.

**Results:**

MASLD mice showed reduced hepatic T lymphocytes, imbalanced CD8^+^/CD4^+^ T ratios, and increased Tregs despite lower CD4^+^ T proportions. Two Treg subsets were identified, one with high CD29 that was overexpressed in HCC; Tregs promoted cell adhesion via CD29 to drive MASH‐HCC transition, and CD29 knockdown attenuated this effect.

**Conclusions:**

Tregs promote cell adhesion through CD29 upregulation, inducing the malignant transformation of MASLD. Targeting CD29 may offer a potential strategy for preventing HCC in patients with MASH.

**Abbreviations**
2‐AAF: 2‐acetylaminofluorene
ALT: alanine aminotransferase
AST: aspartate aminotransferase
CCK‐8: cell counting kit‐8
FOXP3: forkhead box P3
GLU: glucose
GSEA: gene set enrichment analysis
HCC: hepatocellular carcinoma
H&E: hematoxylin and eosin stains
HFD: high fat diet
HPA: human protein atlas
IHC: immunohistochemistry
ITGβ1: integrin beta 1
KEGG: kyoto encyclopedia of genes and genomes
LDL: low‐density lipoprotein
MASH: metabolic dysfunction‐associated steatotic hepatitis
MASLD: metabolic dysfunction‐associated steatotic liver disease
ND: normal diet
TC: total cholesterol
TCGA: The Cancer Genome Atlas
TG: triglycerides

**Summary**

- Tregs enhance cell adhesion via upregulating the expression of CD29, thereby accelerating the malignant progression from MASH to HCC.
- This study emphasises the critical involvement of Tregs with high CD29 expression in the progression of MASLD, suggesting that targeting CD29 to suppress Treg function could represent a viable therapeutic strategy for preventing HCC onset in patients with MASH.

## 1 Introduction

Metabolic dysfunction‐associated steatotic liver disease (MASLD) is the leading chronic liver disease worldwide, particularly prevalent in Western countries, and affects approximately 20% of the global population [1, 2]. MASLD is a disease caused by metabolic alterations resulting from a combination of genetic, dietary and lifestyle factors. Simple steatosis can progress to MASH, with deterioration potentially leading to cirrhosis or even HCC [3, 4]. Currently, there are no effective methods to prevent MASLD, making it critical to explore the underlying pathogenic mechanisms and therapeutic targets involved in its development and progression [5, 6]. Under a healthy liver, the immune system plays a role in surveilling and eliminating abnormal cells, but it can be disrupted by the MASLD microenvironment, leading to immune dysregulation and allowing tumour cells to evade detection [7]. Different T lymphocyte subsets play distinct roles in MASH. CD4^+^ T lymphocytes can inhibit tumour growth via immune surveillance, whereas CD8^+^ T cells are highly enriched in the MASLD microenvironment, contributing to liver inflammation [8, 9, 10]. Tregs, a subset of CD4^+^ T lymphocytes, are characterised by expressing the key transcription factor FoxP3 and are crucial for maintaining immune tolerance and homeostasis. Tregs, as a subset of CD4^+^ T cells, not only fail to be down‐regulated in MASLD but also exhibit increased infiltration [11, 12].

Tregs may serve as potential markers for the progression of MASH to HCC, and targeting Tregs could represent an effective therapeutic strategy for MASH. However, the precise mechanisms underlying their pathogenic potential remain unclear. Integrins are an important class of cell surface transmembrane proteins that are typically composed of heterodimers formed by α and β subunits that mediate cell–cell or cell‐extracellular matrix interactions. Integrins play critical roles in processes such as cell proliferation, migration and signal transduction [13, 14, 15, 16]. CD29, a member of the integrin family, is enriched in various cell types, including immune cells and endothelial cells, where it participates in cell adhesion and inflammatory responses [17, 18]. CD29 promotes liver inflammation in MASH mouse models by mediating cell adhesion, although these studies have focused primarily on the interactions among hepatocytes, monocytes and endothelial cells [19]. The mechanisms involved in the interaction between CD29 and immune cells remain underexplored. Therefore, targeting CD29 to modulate immune cell function may represent a novel and effective therapeutic approach for MASLD. This study investigated the mechanisms by which Tregs promote cell adhesion and induce the progression from MASH to HCC via upregulating CD29. It was demonstrated that targeting CD29 to inhibit the interaction with Tregs is an effective strategy for MASLD malignancy.

## 2 Materials and Methods

### 2.1 Human Samples

The study was approved (ECNU‐2023‐9) by the Ethics Committee of Nantong University and conducted in accordance with the Declaration of Helsinki. Informed consent was obtained from all patients. HCC and adjacent tissues were collected from 10 patients with MASLD, confirmed by Haematoxylin & Eosin staining (H&E) at the Department of Pathology, Affiliated Hospital of Nantong University, China from 2019 to 2024. None of the patients had received chemotherapy or radiotherapy prior to surgery. Liver tissues were immediately frozen in liquid nitrogen for storage until use.

### 2.2 Animals and Animal Models

Six‐week‐old male C57BL/6 mice were purchased from Shanghai Experimental Animal Company (China) and randomly divided into three groups: normal diet (ND) as control, high‐fat diet (HFD) and HFD plus carcinogen group. The HFD group was fed a fat‐rich diet (Shuangshi, D12492, China) composed of 20% crude protein, 20% carbohydrates and 60% crude fat to establish the MASLD model. The HFD plus carcinogen group was fed a 60% HFD and received daily gastric gavage with 25 mg/kg of the hepatocarcinogenic agent 2‐acetylaminofluorene (2‐AAF; Sigma Aldrich, USA) diluted in olive oil to establish the MASH‐HCC mouse model (HCC incidence was 33% at 10–26 weeks and 67% at 22–26 weeks). The mice were housed in a sterile barrier environment (12‐h light–dark cycle, 23°C ± 1°C, 60% relative humidity) with free access to food and water, and daily food intake was monitored. All surgical procedures were approved by the Animal Ethics Committee (P20230224‐009) and performed in a barrier environment at the Experimental Animal Center of Nantong University, China.

### 2.3 Cell Lines

Human liver cell line (THLE2, IMMOCELL, China) and HCC cell lines (HepG2 and LM3) were purchased from YiMo Bio (Cytiva, China). THLE2 cells were cultured in THLE2‐specific culture medium, and HCC cells in DMEM (Cytiva, China) supplemented with 10% fetal bovine serum (FBS, Gibco, USA) in a 37°C incubator with 5% CO_2_. Cell viability was measured using a Cell Counting Kit‐8 (CCK‐8) assay kit (Dojindo, JPN). All the cells tested negative for mycoplasma contamination.

### 2.4 Cell Transfection

When cultured HepG2 and LM3 cells reached 60% to 80% confluency, ITGβ1‐siRNA or control vectors (GenePharma, China) were transfected into HepG2 and LM3 cells via Lipo3000 (Thermo Fisher, USA) and harvested 48 h after transfection, and then their knockdown efficiency was assessed via RT–qPCR.

Other materials and methods are described in the Supporting Information.

## 3 Results

### 3.1 Alterations of Liver Histopathology During MASLD Malignancy

**FIGURE 1:**
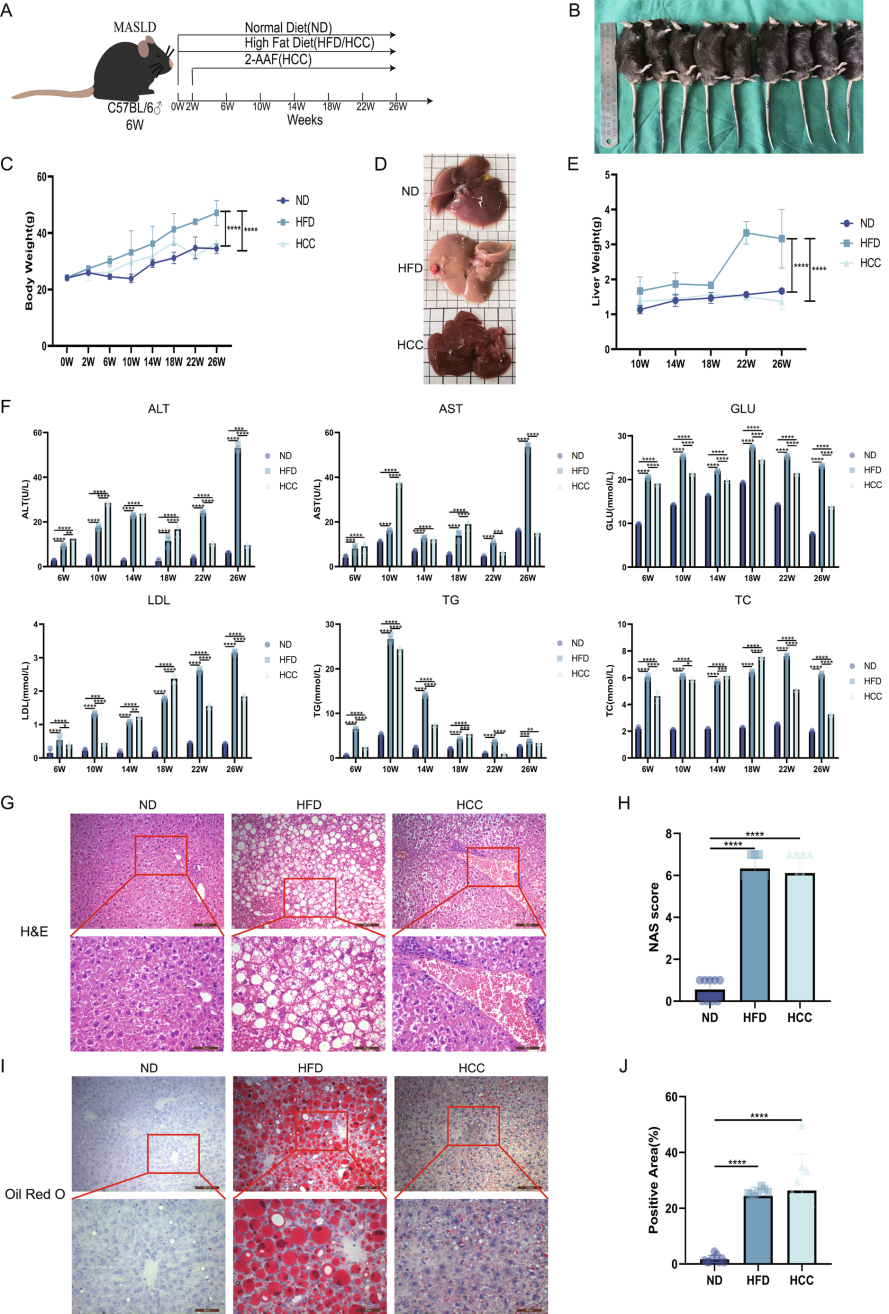
Alterations of Liver Histopathology during MASLD Malignancy. (A) Schematic diagram of the mice MASLD model construction. (B) Representative images of mice from the ND, HFD and HCC groups. (C) Quantification of body weight (*n* = 3/group). (D) Livers of model mice. (E) Liver mass in the ND, HFD and HCC groups (*n* = 3/group). (F) Serum ALT, AST, GLU, LDL, TG and TC levels in MASLD model (*n* = 3/group). (G) H&E staining. (H) NASH activity scores of model livers (*n* = 3/group). (I) Oil Red O staining of mice livers. (J) Quantification of Oil Red O staining (*n* = 3/group). ALT, alanine aminotransferase; AST, aspartate aminotransferase; GLU, glucose; HCC, HFD + carcinogen induction; HFD, high fat diet; LDL, low‐density lipoprotein; ND, normal diet; TC, total cholesterol; TG, triglyceride. *p < 0.05, **p < 0.01, ***p < 0.001 and ****p < 0.0001.

Alterations of liver function, lipids and histopathology during MASLD malignancy are shown in Figure 1. To explore the mechanistic alterations during the progression from normal mice to MASH and subsequent HCC, a MASLD model was established using 6‐week‐old C57BL/6 mice based on the protocol (Figure 1A). Mice were randomised into ND, HFD and HCC groups (Figure 1B) and their model livers (Figure 1C) from MASH to HCC. Images of mouse livers revealed that, in comparison with the ND group, significant lipid accumulation and a marked increase in volume were observed in the HFD group, whereas tumorigenic nodules were detected in the HCC group (Figure 1D). The HFD group showed marked increases (*p* < 0.001) in body weight and liver mass. Conversely, despite rich‐fat feeding, the HCC group exhibited only moderate early‐stage gains in body weight and liver mass due to the cytotoxic effects of the carcinogen, with no significant changes observed in the late disease phase (Figure 1E). Collected sera at distinct time points were assayed for liver function (ALT, AST, *p* < 0.001) and metabolic parameters (GLU, LDL, TG and TC, *p* < 0.001). Both HFD and HCC groups showed stage‐dependent elevations in hepatic and metabolic markers, with a pronounced late‐stage surge in ALT/AST levels indicative of severe hepatic injury and fibrosis in model mice (Figure 1F). Additionally, liver sections were prepared for H&E stained (Figure 1G) with their NASH activity scores (*p* < 0.001, Figure 1H) and Oil Red O stained (Figure 1I) with their positive areas (*p* < 0.001, Figure 1J), revealing extensive steatosis with vacuolar degeneration and fibrous septa in the HFD group. The HCC group displayed additional features of necrotic foci alongside lipid accumulation and fibrosis. Collectively, these findings demonstrated that the MASLD microenvironment induced progressive metabolic dysfunction, hepatic injury, driving inflammatory fibrogenesis and promoting hepato‐carcinogenesis in a subset of mice, as evidenced by HCC formation [20].

### 3.2 Immune Dysfunction of Hepatic T Cells During MASLD Malignancy

**FIGURE 2:**
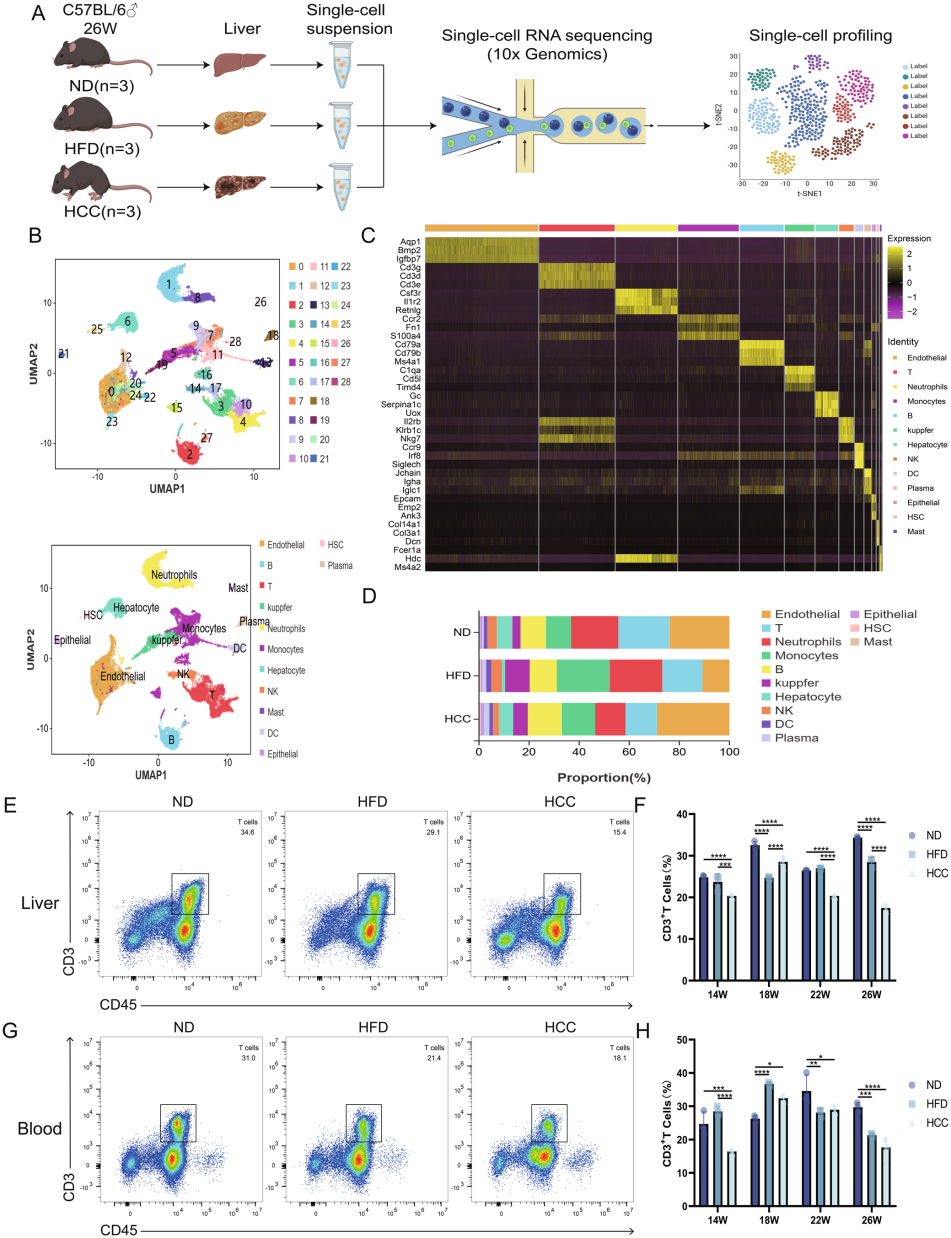
Immune dysfunction of hepatic T cells during MASLD malignancy. (A) Schematic diagram of single‐cell RNA sequencing of liver nonparenchymal cells prepared from MASLD mice (*n* = 3/group). (B) UMAP plot of 24 699 liver nonparenchymal cells from MASLD mice. (C) Heatmap showing the marker genes for each cell subgroup, with three marker genes/subgroup. (D) Proportions of all cell subgroups in the ND, HFD and HCC groups. (E, F) Flow cytometry analysis of liver T cell ratio in ND, HFD and HCC mice at different stages (*n* = 3/group). (G, H) Flow cytometry analysis of peripheral blood T cell proportions in ND, HFD and HCC mice at different stages (*n* = 3/group). HCC, HFD + carcinogen induction; HFD, high‐fat diet; ND, normal diet; UMAP, uniform manifold approximation and projection. *p < 0.05, **p < 0.01, ***p < 0.001, ****p < 0.0001.

Dynamic analysis of hepatic T cell alteration during MASLD malignancy is shown in Figure 2. Mice livers from 26‐week‐old MASLD model (*n* = 9) were enzymatically dissociated into single‐cell suspensions and analysed by scRNA‐seq technology (Figure 2A). After normalisation and clustering of the gene expression matrix, dimensionality reduction was performed using Uniform Manifold Approximation and Projection (UMAP) and a total of 24 699 cells were identified as 29 distinct cell subpopulations. Based on lineage‐specific marker gene expression, these subpopulations were annotated into 13 known cell lineages, including endothelial cells, B and T lymphocytes, neutrophils, monocytes, Kupffer cells, hepatocytes, natural killer (NK) cells, dendritic cells, plasma cells, epithelial cells, hepatic stellate cells (HSCs) and mast cells (Figure 2B). A heatmap depicting the expression of three canonical marker genes per subpopulation is shown (Figure 2C). Quantitative analysis of subpopulation frequencies revealed significant shifts in immune cell proportions, including B, T, Kupffer cells and monocytes [21]. Among these, T cells exhibited the most pronounced dysregulation, with a progressive decline in hepatic abundance throughout disease progression (Figure 2D). To validate these findings, flow cytometry was used to quantify significantly T lymphocytes (*p* < 0.001) in blood, liver and spleen of MASLD mice at distinct stages. Consistent with scRNA‐seq data, both HFD and HCC groups showed reduced T lymphocytes in peripheral blood and livers, with a similar depletion observed in splenic compartments during late‐stage disease (Figure 2E–H, Figure S1A,B) [22]. Collectively, these results demonstrate a profound remodelling of the hepatic immune microenvironment in MASLD, characterised by significant T cell attrition. This immune dysregulation extends beyond the liver to involve systemic compartments such as blood and spleen, highlighting the systemic impact of MASLD‐associated immune dysfunction.

### 3.3 MASLD Microenvironment Modulates T Cell Subpopulation and Function

**FIGURE 3:**
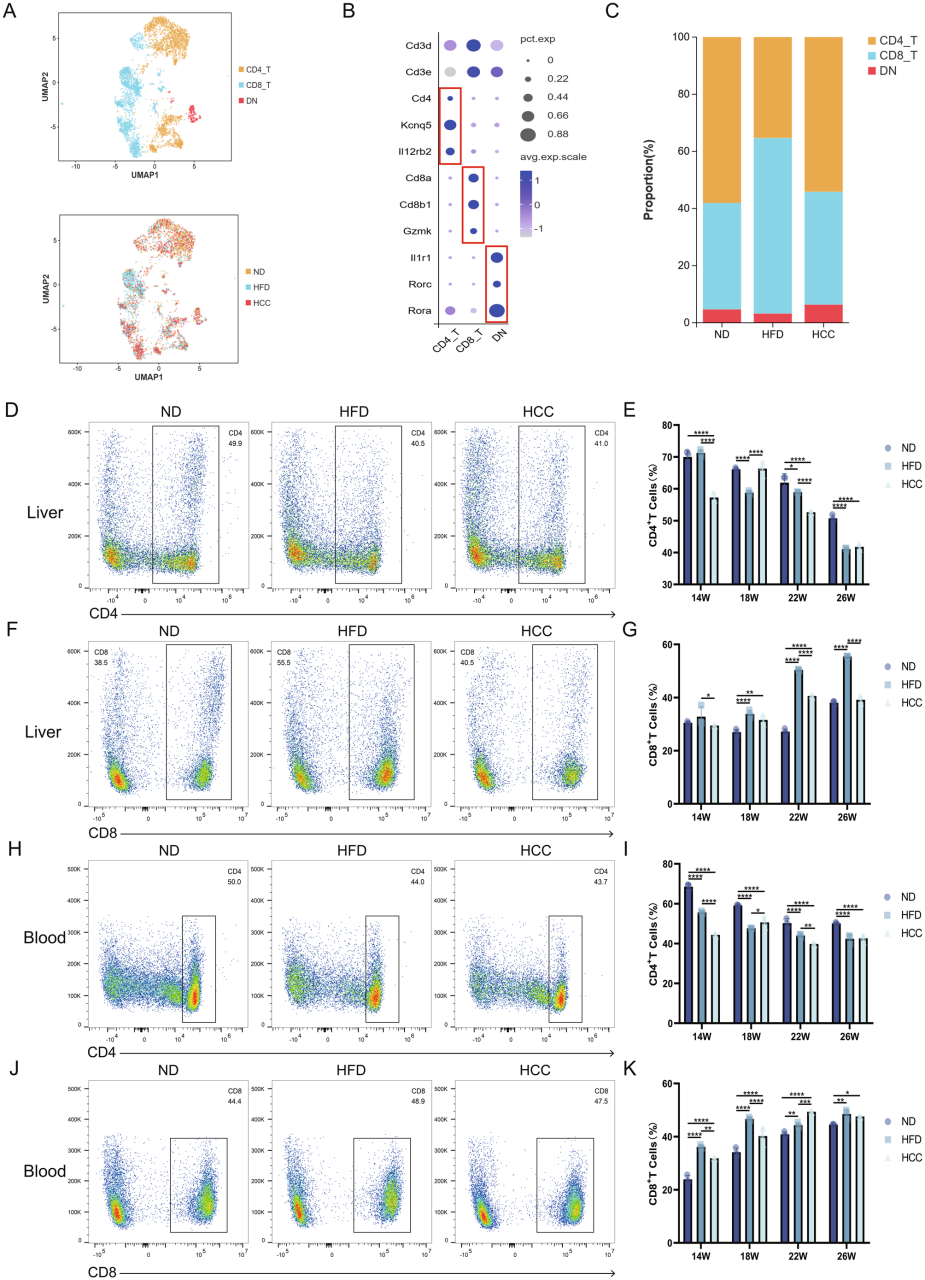
Subpopulation composition of T cells during MASLD malignancy. (A) UMAP plot of T lymphocytes from MASLD model mice, categorised by cell type and sample group. (B) Bubble plot showing marker genes for each T lymphocyte subpopulation, with three marker genes per subpopulation. (C) Proportion of T lymphocyte subpopulations in the ND, HFD and HCC groups. (D–G) Flow cytometry analysis of hepatic CD4^+^ T and CD8^+^ T lymphocyte proportions at different stages in ND, HFD and HCC mice (*n* = 3/group). (H–K) Flow cytometry analysis of CD4^+^ T and CD8^+^ T lymphocyte proportions in peripheral blood at different stages in ND, HFD and HCC mice (*n* = 3/group). HCC, HFD + carcinogen induction; HFD, high‐fat diet; ND, normal diet; UMAP, uniform manifold approximation and projection. *p < 0.05, **p < 0.01, ***p < 0.001, ****p < 0.0001.

To elucidate the dynamic alterations and molecular mechanisms of T lymphocytes in the MASLD microenvironment, GO and KEGG enrichment analyses were performed on T lymphocyte subsets from the three experimental groups. GO enrichment analysis revealed that compared with the ND group, T lymphocytes from both HFD and HCC groups exhibited significant upregulation of immune system processes and leukocyte activation pathways, with prominent enrichment in lymphocyte activation modules, indicating a state of heightened immune activation dominated by lymphocytes in the MASLD environment [22, 23]. KEGG pathway analysis further demonstrated significant enrichment of lipid metabolism and atherosclerosis‐related pathways, consistent with the hyperlipidemic microenvironment characteristic of MASLD [24]. Concomitantly, robust upregulation of T cell receptor (TCR), TNF and NF‐κB signalling was observed, implicating T cell activation and inflammatory cascade initiation (Figure S2A) [25, 26]. Subsequently, T cell subpopulations were phenotypically characterised into CD4^+^ T cells (Cd4, Kcnq5, Il12rb2), CD8^+^ T cells (Cd8a, Cd8b1, Gzmk) and double‐negative (DN) cells (Il1r1, Rorc, Rora, Figure 3A,B). Single‐cell sequencing data showed a stage‐dependent decrease in CD4^+^ T cell counts in HFD and HCC groups, whereas CD8^+^ T cell numbers exhibited variable increases (Figure 3C). These findings were validated by flow cytometric analysis of CD4^+^ and CD8^+^ T cells in peripheral blood, liver and spleen of MASLD model mice at distinct time points. Consistent with scRNA‐seq results, peripheral blood (CD4: 14 W: ND: 66.8%–69.5%, HFD: 55.1%–56.3%, HCC: 43.5%–45.1%, ND vs. HFD: *p* < 0.0001, ND vs. HCC: *p* < 0.0001, HFD vs. HCC: *p* < 0.0001, 18 W: ND: 59%–59.5%, HFD: 47.4%–47.8%, HCC: 47.9%–52.9%, ND vs. HFD: *p* < 0.0001, ND vs. HCC: *p* < 0.0001, HFD vs. HCC: *p* = 0.042, 22 W: ND: 48%–52.6%, HFD: 43%–44.7%, HCC: 39.4%–40.3%, ND vs. HFD: *p* < 0.0001, ND vs. HCC: *p* < 0.0001, HFD vs. HCC: *p* < 0.0036, 26 W: ND: 49.7%–50.6%, HFD: 39.8%–44%, HCC: 40.7%–43.7%, ND vs. HFD: *p* < 0.0001, ND vs. HCC: *p* < 0.0001, HFD vs. HCC: *p* = 0.9641, CD8: 14 W: ND: 22.8%–25.6%, HFD: 35.6%–37%, HCC: 31.7%–31.9%, ND vs. HFD: *p* < 0.0001, ND vs. HCC: *p* < 0.0001, HFD vs. HCC: *p* = 0.0012, 18 W: ND: 32.7%–35.9%, HFD: 46%–47.3%, HCC: 37%–43%, ND vs. HFD: *p* < 0.0001, ND vs. HCC: *p* < 0.0001, HFD vs. HCC: *p* < 0.0001, 22 W: ND: 40%–41.9%, HFD: 43.5%–45.4%, HCC: 49.1%–49.7%, ND vs. HFD: *p* = 0.0086, ND vs. HCC: *p* < 0.0001, HFD vs. HCC: *p* = 0.0002, 26 W: ND: 44.4%–44.7%, HFD: 46.6%–50%, HCC: 47.4%–47.8%, ND vs. HFD: *p* = 0.0027, ND vs. HCC: *p* = 0.0223, HFD vs. HCC: *p* = 0.6563) and liver (CD4: 14 W: ND: 67.6%–71.5%, HFD: 70.2%–72.3%, HCC: 56.4%–58.8%, ND vs. HFD: *p* = 0.5022, ND vs. HCC: *p* < 0.0001, HFD vs. HCC: *p* < 0.0001, 18 W: ND: 65.6%–66.6%, HFD: 58.2%–59.4%, HCC: 64.1%–68.2%, ND vs. HFD: *p* < 0.0001, ND vs. HCC: *p* = 0.9762, HFD vs. HCC: *p* < 0.0001, 22 W: ND: 58.6%–63.6%, HFD: 58.8%–59.1%, HCC: 52.5%–52.8%, ND vs. HFD: *p* = 0.0331, ND vs. HCC: *p* < 0.0001, HFD vs. HCC: *p* < 0.0001, 26 W: ND: 49.9%–51.9%, HFD: 40.5%–41.4%, HCC: 41%–42.7%, ND vs. HFD: *p* < 0.0001, ND vs. HCC: *p* < 0.0001, HFD vs. HCC: *p* = 0.79, CD8: 14 W: ND: 30.2%–31%, HFD: 30.4%–37.1%, HCC: 29.3%–29.7%, ND vs. HFD: *p* = 0.1289, ND vs. HCC: *p* = 0.6664, HFD vs. HCC: *p* = 0.0213, 18 W: ND: 25.8%–28.1%, HFD: 32.6%–35.3%, HCC: 30%–33.4%, ND vs. HFD: *p* < 0.0001, ND vs. HCC: *p* = 0.0014, HFD vs. HCC: *p* = 0.151, 22 W: ND: 26.4%–28.3%, HFD: 50%–50.7%, HCC: 40.1%–40.8%, ND vs. HFD: *p* < 0.0001 ND vs. HCC: *p* < 0.0001, HFD vs. HCC: *p* < 0.0001, 26 W: ND: 37.1%–38.5%, HFD: 55.3%–55.6%, HCC: 37.9%–40.5%, ND vs. HFD: *p* < 0.0001, ND vs. HCC: *p* = 0.5954, HFD vs. HCC: *p* < 0.0001) samples showed time‐dependent declines in CD4^+^ T cells and concurrent elevations in CD8^+^ T cells. In splenic compartments (CD4: 14 W: ND: 60.9%–63.3%, HFD: 55.2%–62.2%, HCC: 57.5%–59.5%, ND vs. HFD: *p* = 0.0631, ND vs. HCC: *p* = 0.157, HFD vs. HCC: *p* = 0.8843, 18 W: ND: 56%–56.3%, HFD: 53.3%–54.1%, HCC: 52.7%–53.9%, ND vs. HFD: *p* = 0.4487, ND vs. HCC: *p* = 0.2891, HFD vs. HCC: *p* = 0.9466, 22 W: ND: 60.2%–60.7%, HFD: 52.5%–54.1%, HCC: 59.3%–62.3%, ND vs. HFD: *p* = 0.0025, ND vs. HCC: *p* = 0.9073, HFD vs. HCC: *p* = 0.0009, 26 W: ND: 54.4%–58.8%, HFD: 38.4%–49.3%, HCC: 40.4%–43.8%, ND vs. HFD: *p* < 0.0001, ND vs. HCC: *p* < 0.0001, HFD vs. HCC: *p* = 0.382, CD8: 14 W: ND: 30.2%–33.6%, HFD: 23.7%–25.8%, HCC: 28.5%–29.6%, ND vs. HFD: *p* < 0.0001, ND vs. HCC: *p* = 0.0036, HFD vs. HCC: *p* = 0.0002, 18 W: ND: 36.4%–37%, HFD: 38.1%–38.9%, HCC: 37.5%–39.5%, ND vs. HFD: *p* = 0.0976, ND vs. HCC: *p* = 0.1052, HFD vs. HCC: *p* = 0.9992, 22 W: ND: 33.6%–34.7%, HFD: 30.9%–31.8%, HCC: 33.3%–34.3%, ND vs. HFD: *p* = 0.0063, ND vs. HCC: *p* = 0.7223, HFD vs. HCC: *p* = 0.0375, 26 W: ND: 34.2%–36.4%, HFD: 33.4%–37.9%, HCC: 33.8%–34.1%, ND vs. HFD: *p* = 0.9604, ND vs. HCC: *p* = 0.1615, HFD vs. HCC: *p* = 0.0976), CD8^+^ T cell numbers remained unchanged, while CD4^+^ T cell counts decreased specifically in late‐stage disease (figure 3D‐K, S1C‐F) [8, 9, 10]. Collectively, these data suggest that the MASLD microenvironment induces compartment‐specific remodelling of T cell subpopulations in peripheral blood, liver and spleen, leading to functional reprogramming that may contribute to immune dysregulation and disease progression.

### 3.4 Proliferation, Adhesion and Migration of Tregs via Microenvironment

Subgroup analysis was performed on the markedly expanded CD8^+^ T‐cell populations in the MASLD model, and eight subsets were identified, with particular emphasis placed on the predominant groups: naïve CD8^+^ T cells (Sell, Lef1, Tcf7), central memory CD8^+^ T cells(Trbc1, Cdk6, Adam19), terminal effector memory CD8^+^ T cells (Il18rap, Cd29, Rap1gap2) and exhausted CD8^+^ T cells (Havcr2, Ctla4, Pdcd1, Figure S3A,B). Compared with the ND group, both central memory and terminal effector memory CD8^+^ T cells were significantly increased in the HFD group, indicating that cytotoxic CD8^+^ T‐cell subsets with antitumour activity were continuously induced during the progression of MASLD. However, the proportion of exhausted CD8^+^ T cells was elevated, suggesting that tumoricidal CD8^+^ T cells were overactivated and subsequently driven into functional exhaustion, characterised by a marked reduction in cytotoxicity and proliferative capacity. In the HCC group, central memory and terminal effector memory CD8^+^ T cells were decreased, reflecting the depletion of cytotoxic effector populations and the attenuation of antitumour activity. Meanwhile, exhausted CD8^+^ T cells were also reduced, which was associated with the loss of proliferative and differentiation potential and the progression toward apoptosis. The loss of these functional CD8^+^ T cells consequently resulted in a relative increase in the proportion of naïve CD8^+^ T cells, whose absolute numbers otherwise remained stable (Figure S3C) [27, 28, 29]. Given the unique role of CXCR6^+^ CD8^+^ T cells in anti‐infective and antitumour immunity [30], CXCR6 expression was further examined. No significant difference was observed between the HFD and ND groups, whereas a marked decline was detected in the HCC group (Figure S3D). Given the exhaustion of functional CD8^+^ T cell populations, it is hypothesised that the functional impairment of CD8^+^ T cells is intensified in the advanced stage of the disease, and their antitumour efficacy is weakened, thereby contributing to the further progression of the disease.

**FIGURE 4:**
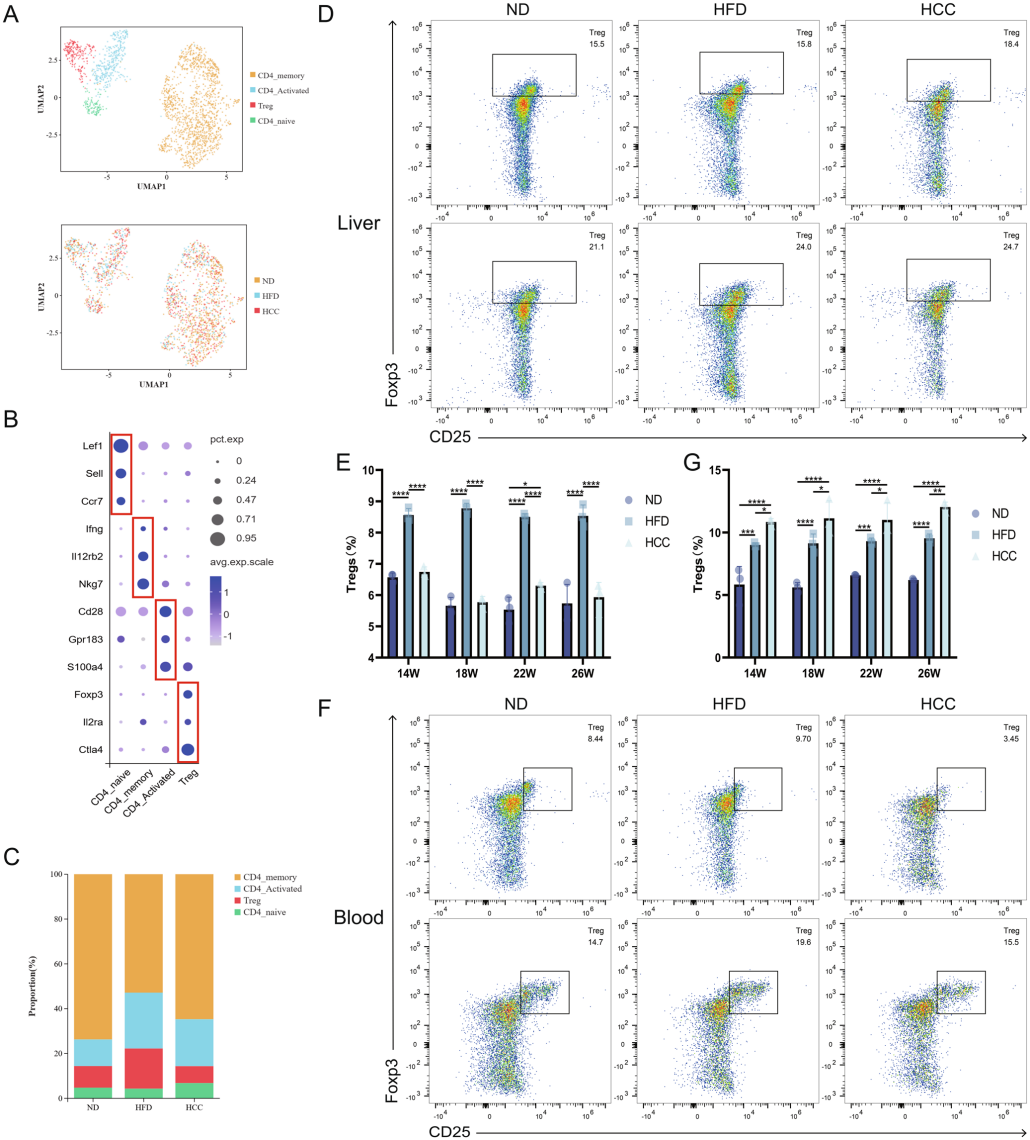
Composition of Tregs in MASLD model mice. (A) UMAP plot of CD4^+^ T lymphocytes from MASLD model mice, categorised by cell type and sample group. (B) Bubble plot showing marker genes for CD4^+^ T lymphocyte subpopulations, with three marker genes for each subpopulation. (C) Proportions of CD4^+^ T lymphocyte subpopulations in the ND, HFD and HCC groups. (D, E) Flow cytometry analysis of hepatic Treg proportions at different stages in ND, HFD and HCC mice (*n* = 3/group). (F, G) Flow cytometry analysis of peripheral blood Treg proportions at different stages in ND, HFD and HCC mice (*n* = 3/group). HCC, HFD + carcinogen induction; HFD, high‐fat diet; ND, normal diet. *p < 0.05, **p < 0.01, ***p < 0.001, ****p < 0.0001.

Focusing on the significantly reduced CD4^+^ T cells in MASLD model mice (Figure 4), subpopulation profiling classified them into CD4 memory T cells (Ifng, Il12rb2, Nkg7), activated CD4^+^ T cells (Cd28, Gpr183, S100a4), naive CD4^+^ T cells (Lef1, Sell, Ccr7) and Tregs (Foxp3, Il2ra, Ctla4, Figure 4A,B). Quantitative analysis of subpopulation frequencies by single‐cell sequencing showed a significant increase in hepatic Tregs in the HFD group compared with ND controls, whereas no change was observed in the HCC group (Figure 4C). Flow cytometric analysis of Tregs in peripheral blood (14 W: ND: 4.2%–7%, HFD: 8.9%–9.2%, HCC: 10.5%–11%, ND vs. HFD: *p* = 0.0001, ND vs. HCC: *p* < 0.0001, HFD vs. HCC: *p* = 0.0209, 18 W: ND: 5.2%–6%, HFD: 8.4%–9.9%, HCC: 10%–12.7%, ND vs. HFD: *p* < 0.0001, ND vs. HCC: *p* < 0.0001, HFD vs. HCC: *p* = 0.0114, 22 W: ND: 6.5%–6.6%, HFD: 9.1%–9.6%, HCC: 10.2%–12.6%, ND vs. HFD: *p* = 0.0007, ND vs. HCC: *p* < 0.0001, HFD vs. HCC: *p* = 0.0334, 26 W: ND: 6.1%–6.3%, HFD: 9.2%–9.9%, HCC: 11.6%–12.4%, ND vs. HFD: *p* < 0.0001, ND vs. HCC: *p* < 0.0001, HFD vs. HCC: *p* = 0.0017), liver (14 W: ND: 6.4%–6.7%, HFD: 8.4%–8.8%, HCC: 6.6%–6.9%, ND vs. HFD: *p* < 0.0001, ND vs. HCC: *p* = 0.8583, HFD vs. HCC: *p* < 0.0001, 18 W: ND: 5.4%–6%, HFD: 8.6%–8.9%, HCC: 5.6%–5.9%, ND vs. HFD: *p* < 0.0001, ND vs. HCC: *p* = 0.8583, HFD vs. HCC: *p* < 0.0001, 22 W: ND: 5.1%–5.9%, HFD: 8.4%–8.6%, HCC: 6.2%–6.4%, ND vs. HFD: *p* < 0.0001, ND vs. HCC: *p* = 0.0151, HFD vs. HCC: *p* < 0.0001, 26 W: ND: 5.2%–6.4%, HFD: 8.2%–8.9%, HCC: 5.4%–6.3%, ND vs. HFD: *p* < 0.0001, ND vs. HCC: *p* = 0.7112, HFD vs. HCC: *p* < 0.0001) and spleen (14 W: ND: 6.1%–8%, HFD: 7.9%–8.2%, HCC: 14%–14.3%, ND vs. HFD: *p* = 0.0646, ND vs. HCC: *p* < 0.0001, HFD vs. HCC: *p* < 0.0001, 18 W: ND: 9.3%–9.7%, HFD: 9.4%–10.8%, HCC: 8.9%–10.6%, ND vs. HFD: *p* = 0.3789, ND vs. HCC: *p* = 0.9603, HFD vs. HCC: *p* = 0.5321, 22 W: ND: 8.1%–10.3%, HFD: 9.4%–10.3%, HCC: 10%–11%, ND vs. HFD: *p* = 0.6149, ND vs. HCC: *p* = 0.0561, HFD vs. HCC: *p* = 0.3122, 26 W: ND: 17.4%–17.8%, HFD: 14.9%–15.7%, HCC: 16.4%–16.7%, ND vs. HFD: *p* = 0.0003, ND vs. HCC: *p* = 0.1422, HFD vs. HCC: *p* = 0.0314) of MASLD model mice revealed concordance with scRNA‐seq data in the liver, demonstrating HFD‐induced Treg expansion without HCC‐stage changes. In contrast, peripheral blood Tregs trended upward in both HFD and HCC groups, while splenic Tregs showed no clear pattern (Figure 4D–G, Figure S4A,B) [11, 12]. This distinct peripheral blood Treg surge in HCC mice suggests that during MASH‐to‐HCC progression, liver‐resident Tregs undergo activation‐driven functional reprogramming, promoting their egress from the hepatic niche into the systemic circulation.

To validate this hypothesis, CellChat was employed to quantify intercellular communication between Tregs and other lineages across groups. Compared with ND mice, HFD and HCC groups exhibited significantly increased communication intensity between Tregs and endothelial cells (Figure S5A). Given the adhesive and migratory properties of endothelial cells, this crosstalk was hypothesised to enhance Treg adhesion and migration [31, 32, 33]. Receptor‐ligand bubble plot analysis further revealed that cell adhesion‐related ligand‐receptor pairs (e.g., integrin family and cadherins) were markedly upregulated in the HCC group Treg‐endothelial cell interactions, among which the communication intensity of ITGB1 and its ligand JAM2 was the most prominent. This suggests that during the transition from MASH to HCC, ITGB1 enhances the motility of regulatory T cells by binding to ligands such as JAM2 (Figure S5B). Cytokine expression profiling of CD4^+^ T cell subsets showed significant upregulation of vascular endothelial growth factor A (VEGFA) in the HCC group. As a key mediator of angiogenesis and endothelial cell proliferation, VEGFA promotes Treg migration and tissue invasion [34]. The VEGFA upregulation underscores the functional crosstalk between Tregs and endothelial cells, corroborating enhanced Treg adhesive and migratory capacities. Concomitantly, chemokine‐related genes (e.g., CXCR4, CXCL2) were transcriptionally upregulated in both HFD and HCC groups (Figure S5C) [35]. Given that endothelial cells drive the MASH‐to‐HCC transition via the promotion of cell adhesion and migration [19, 36], these findings collectively indicate that Treg‐endothelial cell interaction potentiates Treg clonal expansion and motility, enabling their dissemination from hepatic parenchyma to systemic circulation. This mechanism may explain the observed enrichment of Tregs in peripheral blood and spleen relative to the liver in HCC mice.

### 3.5 Phenotypic Characterisation of Treg Subsets and Pathogenic Mechanisms

**FIGURE 5:**
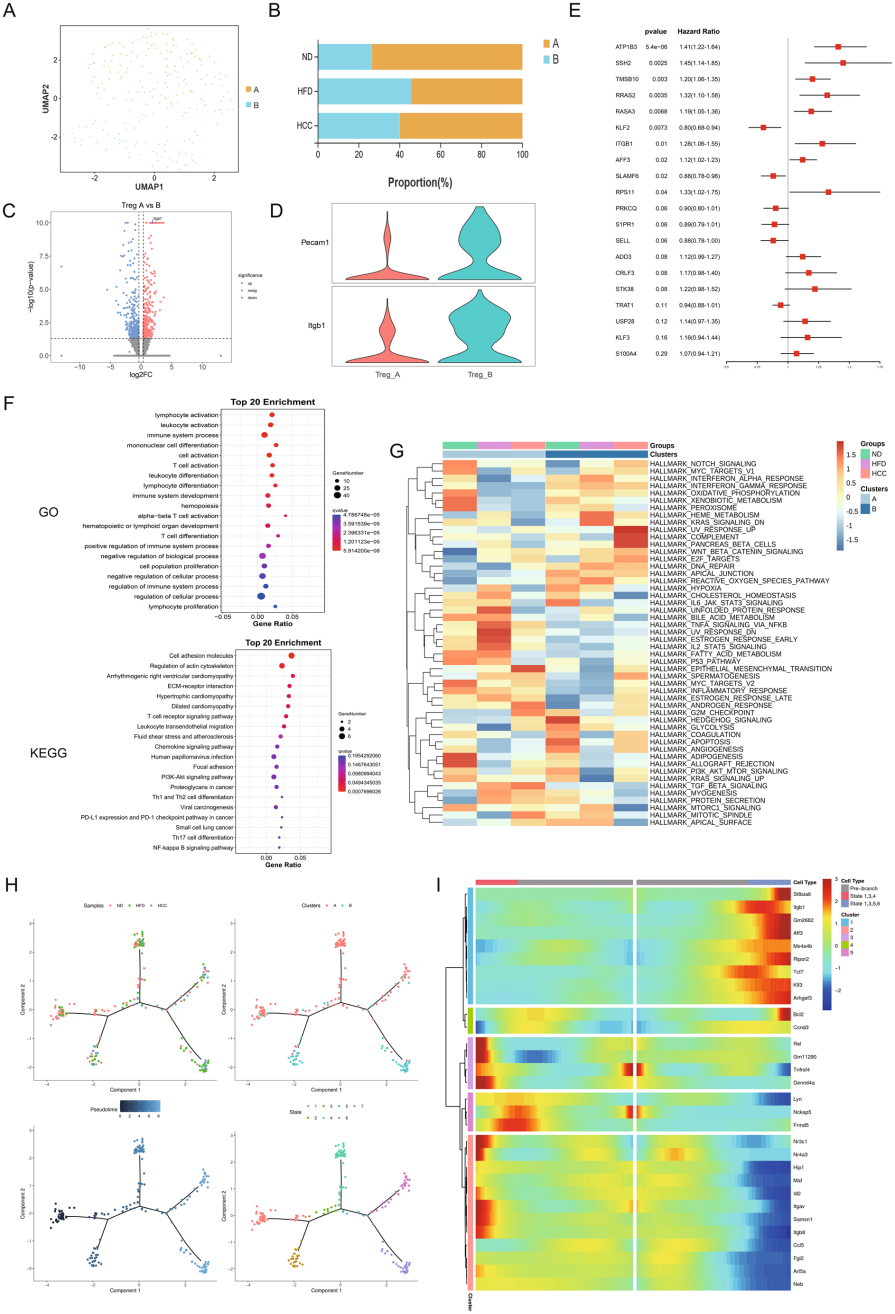
Tregs subtype identification and their pathogenic mechanisms. (A) UMAP dimensionality reduction plot of Tregs from MASLD model mice obtained through unsupervised clustering. (B) Proportions of Treg subpopulations in the ND, HFD and HCC groups. (C) Volcano plot of differentially expressed genes in Tregs subpopulations. (D) Violin plots of Treg subpopulations. (E) Forest plot of univariate regression analysis of upregulated genes in Treg B cells. (F) GO and KEGG enrichment analyses of Treg subpopulations. (G) Heatmap of the results of GSVA analysis of Tregs subpopulations. (H) Pseudotime analysis trajectory of Tregs, categorised by sample, cell subpopulation, pseudotime and state. (I) Branch analysis of Treg states 1, 3 and 4 and states 1, 3, 5 and 6. GSVA, gene set variation analysis; HCC, HFD + carcinogen induction; HFD, high‐fat diet; ND, normal diet; UMAP, uniform manifold approximation and projection. *p < 0.05, **p < 0.01, ***p < 0.001, ****p < 0.0001.

Phenotypic characterisation of Treg subsets and pathogenic mechanisms is shown in Figure 5. To further dissect the pathogenic mechanisms of Tregs in MASLD, unsupervised clustering analysis was employed to phenotypically classify Tregs into two distinct subpopulations: Treg A and Treg B (Figure 5A). Quantitative immunophenotyping revealed a significant enrichment of Treg A cells in the ND group, whereas Treg B cells exhibited preferential expansion in HFD and HCC cohorts. This observation led to the hypothesis that Treg B cells assume a dominant pathogenic role in MASLD progression (Figure 5B). A volcano plot was constructed to visualise differentially expressed genes (DEGs) between Treg A and Treg B subpopulations, with a focus on Treg B‐upregulated transcripts. Notably, CD29 was identified as a significantly upregulated gene in Treg B cells (Figure 5C). As a member of the integrin family, CD29 functions as a cell adhesion molecule implicated in MASLD pathogenesis and demonstrates high expression in endothelial cells [19]. Concurrently, CD29 and PECAM1—two genes associated with endothelial cell biology and cell adhesion—were selected from Treg B‐upregulated DEGs, and a violin plot was generated to validate expression differences. The data showed significantly elevated CD29 and PECAM1 expression in Treg B versus Treg A cells, suggesting that PECAM1 may cooperate with CD29 to enhance the adhesion and migratory capacity of the Treg B subpopulation, rendering it a pathogenic subset (Figure 5D) [37]. Univariate Cox regression analysis was performed on Treg B‐upregulated genes to calculate risk scores, identifying the top 20 risk‐associated genes.

Notably, CD29 was re‐identified as a high‐risk scoring gene, reinforcing its potential as a key pathogenic mediator in Treg B cells (Figure 5E). Functional enrichment analyses (GO and KEGG) of DEGs across Treg subpopulations were subsequently conducted. GO analysis revealed significant enrichment of lymphocyte activation and immune system process pathways, indicative of heightened Treg activation [22, 23]. KEGG analysis further identified overrepresentation of cell adhesion molecule and ECM‐receptor interaction pathways, suggesting enhanced adhesive and migratory properties of Tregs (Figure 5F) [38, 39, 40]. Gene set variation analysis (GSVA) of Tregs across the three groups revealed significant upregulation of cancer‐related pathways—including WNT‐β‐catenin, E2F and epithelial‐mesenchymal transition (EMT) signalling—in HFD and HCC groups. This suggests that Tregs within the MASLD microenvironment activate oncogenic pathways to promote cellular proliferation and migration (Figure 5G) [41, 42, 43]. Additionally, metabolic activity profiling using the scMetabolism package demonstrated significant downregulation of fatty acid degradation and other metabolic pathways in HFD/HCC groups, indicating profound metabolic reprogramming of Tregs in the MASLD microenvironment (Figure S6A).

Pseudotime trajectory analysis of Tregs across all groups categorised developmental states into seven distinct phases. Treg A cells predominated at the initial developmental nodes, with branching trajectories leading to self‐renewal or differentiation toward Treg B. Treg A cells were primarily localised in States 1, 3, 4, and branches of States 1, 3, 5, 7, whereas Treg B cells were enriched in States 1, 2, and branches of States 1, 3, 5, 6. ND group Tregs primarily occupied early to middle developmental stages (branches of States 1, 3, 4), while pathological HFD/HCC Tregs were skewed toward late stage development (branches of States 1, 3, 5, 6 and 1, 3, 5, 7) (Figure 5H). Pseudotime‐dependent upregulated genes were predominantly associated with cell adhesion (Figure S6B). Branch‐specific analysis comparing ND group‐dominant branches (States 1, 3, 4) and HFD/HCC‐dominant branches (States 1, 3, 5, 6 and 1, 3, 5, 7) revealed upregulation of pathogenicity‐related genes—including CD29—in pathological branches (Figure 5I). Collectively, these findings identify Treg B cells—characterised by high CD29 expression—as a major pathogenic subpopulation within Tregs. It is proposed that Treg B cells promote cellular adhesion and migration, thereby facilitating the transition from MASLD to HCC.

### 3.6 Tregs Drive MASLD Malignancy via Upregulating CD29

**FIGURE 6:**
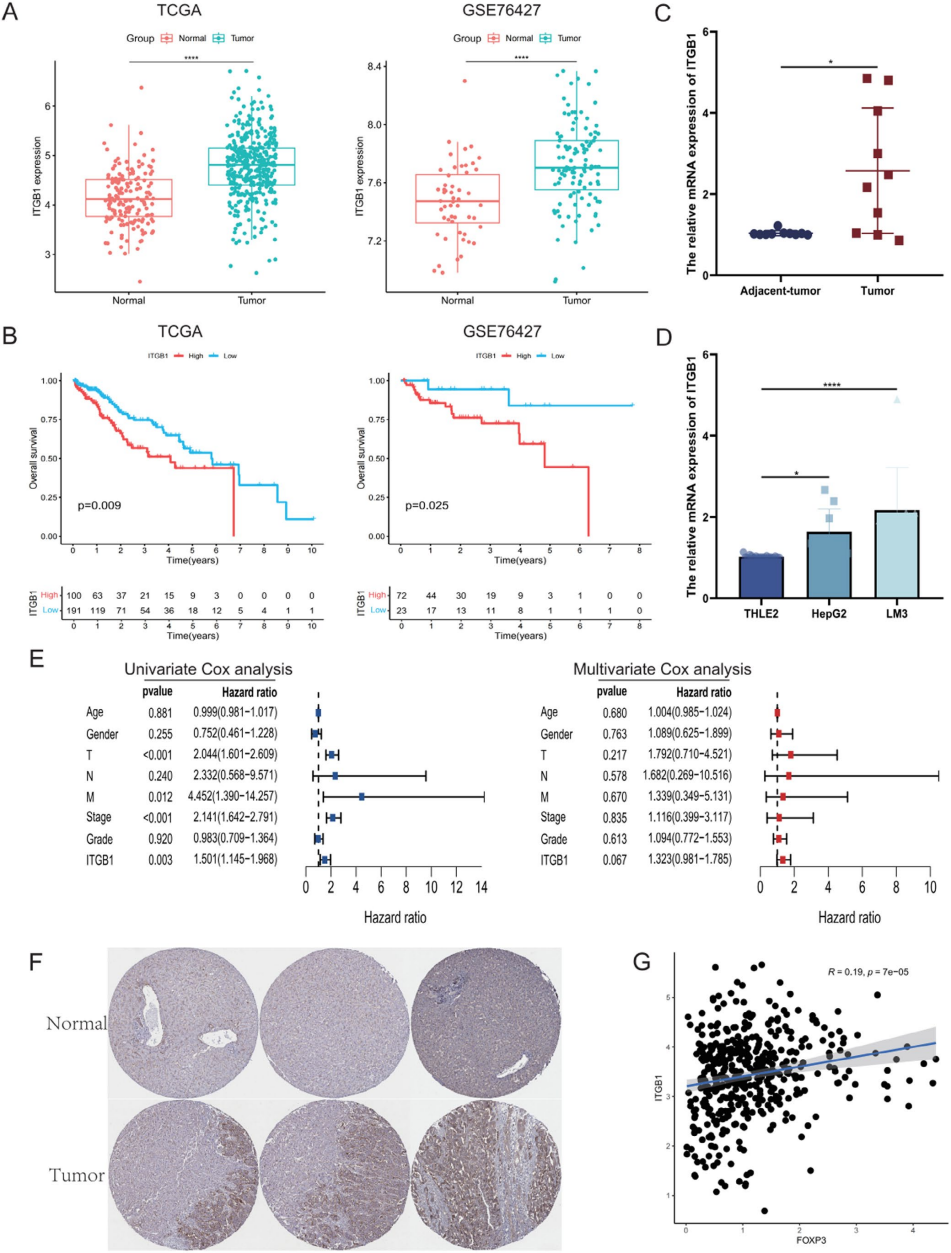
Upregulated CD29 in HCC patients with poor prognosis. (A) Validation of CD29 expression levels via data from the GEO and TCGA databases. (B) Verification of clinical prognosis outcomes associated with CD29 in the GEO and TCGA databases. (C) Assessment of CD29 at mRNA level in adjacent nontumor and tumour tissues from HCC patients (*n* = 10). (D) Validation of CD29 at mRNA levels in HCC cell lines. (E) Univariate and multivariate regression analysis forest plots correlating the clinical information of HCC patients from TCGA database. (F) Immunohistochemical analysis of CD29 in healthy individuals and HCC patients as shown in the HPA database. (G) Pearson correlation analysis of CD29 and Foxp3 expression in TCGA database. GEO, gene expression omnibus; HPA, human protein atlas; TCGA, The Cancer Genome Atlas. *p < 0.05, **p < 0.01, ***p < 0.001, ****p < 0.0001.

Tregs drive MASLD malignancy via upregulating CD29 as shown in Figure 6. Using data from the GEO and TCGA databases, we demonstrated elevated CD29 expression in HCC patients. Clinical prognostic analysis further confirmed that patients with high CD29 expression exhibited poorer survival outcomes (Figure 6A,B). qPCR of clinical samples from HCC patients validated CD29 upregulation in MASH‐HCC cases (*p* = 0.0124, Figure 6C). Compared to the normal human liver cell line THLE2, both HepG2 and LM3 hepatoma cell lines showed significant CD29 overexpression, with LM3 demonstrating the highest levels (*p* < 0.001, Figure 6D). Compilation of TCGA‐derived HCC patient data followed by univariate and multivariate Cox regression analyses identified CD29 as an independent risk factor in both models (Table S1, Figure 6E). Querying the HPA confirmed elevated CD29 expression in HCC tissues versus healthy controls (Figure 6F). Correlation analysis revealed a positive association between CD29 and FOXP3 in HCC patients, suggesting a mechanistic link whereby CD29^+^ Tregs promote HCC progression via CD29 upregulation. (*R* = 0.19, *p* < 0.001, Figure 6G).

**FIGURE 7:**
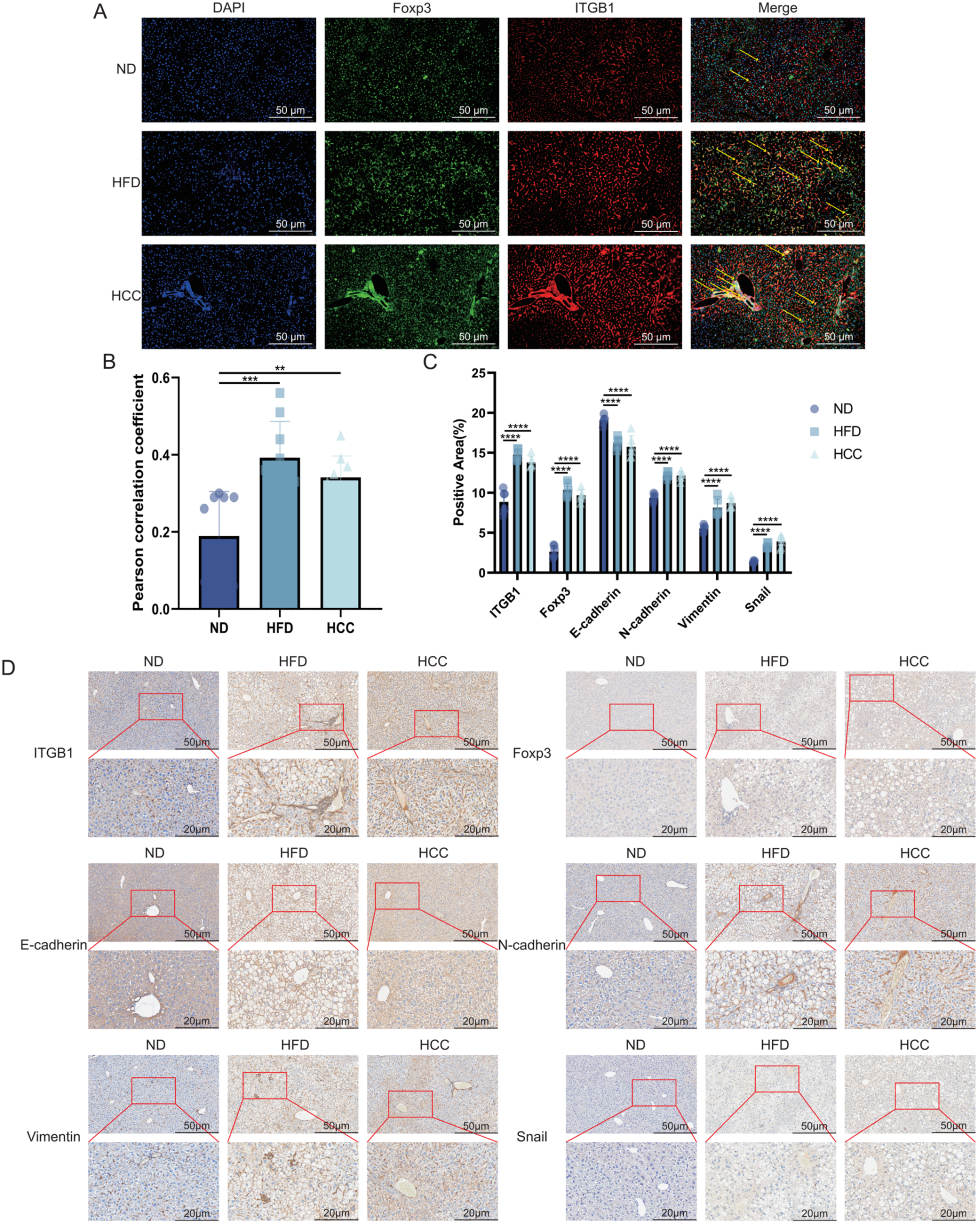
CD29 Promoted EMT via Interaction with Foxp3 in MASLD. (A, B) Immunofluorescence colocalization and correlation analysis of CD29 and Foxp3 in MASLD model mice. (C, D) Immunohistochemistry analysis of CD29, Foxp3 and EMT‐related genes in MASLD model mice. *p < 0.05, **p < 0.01, ***p < 0.001, ****p < 0.0001.

In liver tissues from MASLD model mice, immunofluorescence staining confirmed enhanced CD29‐FOXP3 colocalization within the MASLD microenvironment (Figure 7). Compared to the ND group, both HFD and HCC groups exhibited significantly increased CD29‐FOXP3 co‐expression, indicating strengthened Treg‐CD29 interaction in pathological states. (*p* < 0.001, Figure 7A,B). Immunohistochemistry of liver tissues from MASLD model mice showed that, compared with the ND group, CD29 and FOXP3 were concurrently upregulated in the HFD and HCC groups, accompanied by enhanced EMT markers, with no significant differences observed between the HFD and HCC groups (*p* < 0.001, Figure 7C,D). Although the positive areas of CD29, FOXP3 and EMT markers in the HCC group were not noticeably lower than those in the HFD group, all were significantly increased compared with the ND group, largely consistent with flow cytometry and single‐cell sequencing results. These findings establish a significant correlation between Tregs and CD29 in the MASLD microenvironment, demonstrating their coordinated overexpression. The data support a pathogenic model wherein Tregs promote cellular adhesion and drive MASH‐to‐HCC progression via CD29‐mediated mechanisms.

### 3.7 CD29 Knockdown Suppresses MASLD Malignancy

**FIGURE 8:**
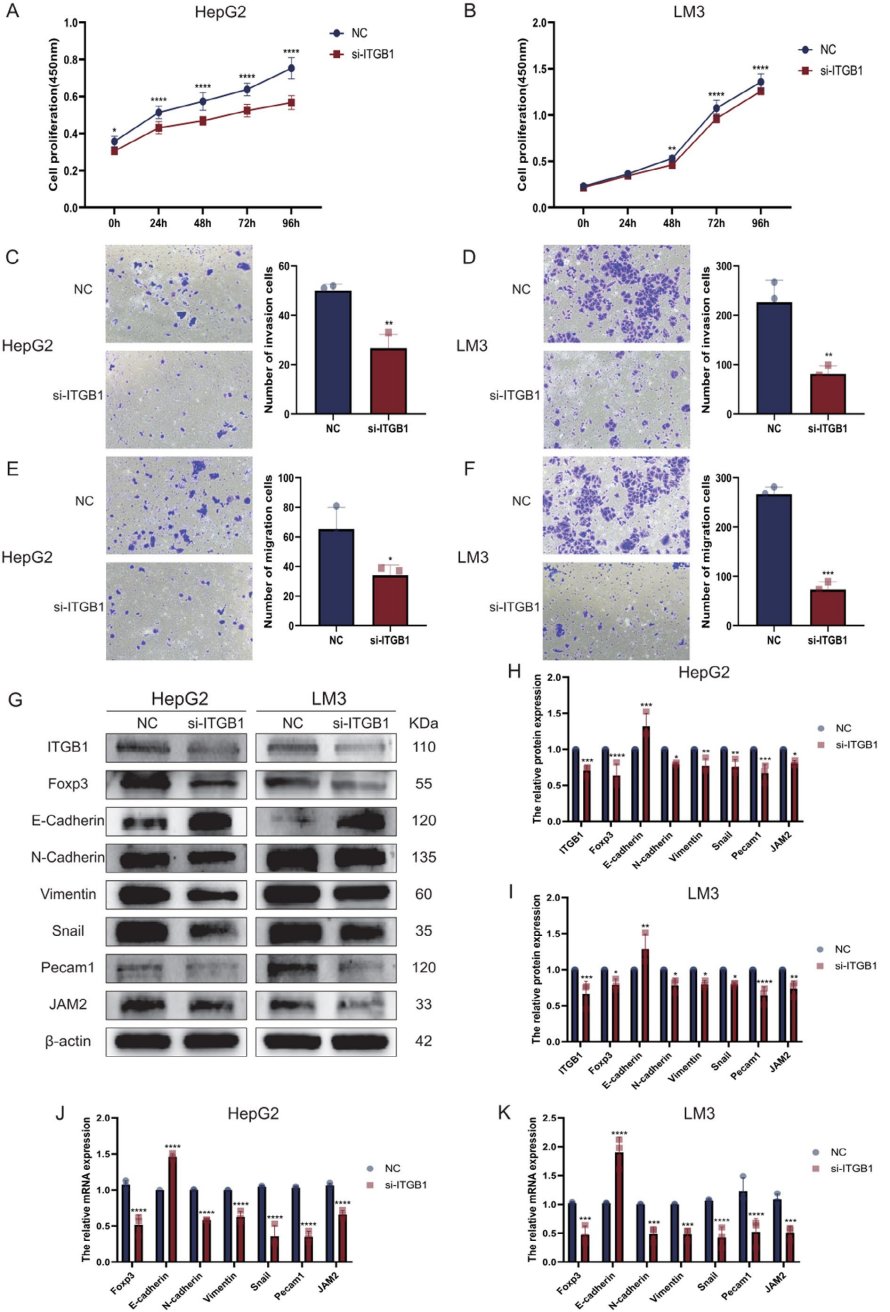
Knockdown of CD29 inhibited the biological behaviours of HCC cells. (A, B) CCK‐8 assay verifying the proliferative capacity of HepG2 and LM3 cells. (C–F) Transwell assay validating the invasion and migration abilities of HepG2 and LM3 cells. (G–I) Western blot (WB) analysis of the protein expression levels of CD29 and its associated ligands, Foxp3 and EMT markers in HepG2 and LM3 cells. (J, K) qPCR was used to assess CD29 at mRNA level and its related ligands, Foxp3 and EMT markers in HepG2 and LM3 cells. CCK‐8, cell counting kit‐8; EMT, epithelial–mesenchymal transition; qPCR, quantitative polymerase chain reaction; WB, western blot. *p < 0.05, **p < 0.01, ***p < 0.001, ****p < 0.0001.

To characterise the pathogenic role of CD29 in MASH progression, small interfering RNA (siRNA)‐mediated CD29 knockdown was performed in HepG2 and LM3 hepatoma cell lines (Figure 8). CCK‐8 assays revealed significant reductions in proliferative capacity following CD29 silencing in both cell lines (HepG2: *p* < 0.001, LM3: *p* < 0.001, Figure 8A,B). Transwell invasion and migration assays further demonstrated that CD29 depletion markedly attenuated invasive (HepG2: *p* = 0.0027, LM3: *p* = 0.006) and migratory (HepG2: *p* = 0.0287, LM3: *p* < 0.001) capabilities (Figure 8C–F). qPCR and Western blot analyses of CD29‐knockdown cells showed coordinated down‐regulation of FOXP3 at both transcriptional (HepG2: *p* < 0.001, LM3: *p* < 0.001) and translational (HepG2: *p* < 0.001, LM3: *p* < 0.001) levels, accompanied by reduced EMT marker expression and attenuated integrin receptor signalling (Figure 8G–K). These findings validate that CD29 depletion disrupts Treg‐mediated adhesive crosstalk, thereby inhibiting malignant progression from MASH to HCC. Collectively, the data establish a pathogenic model wherein Tregs undergo phenotypic reprogramming within the MASLD microenvironment, driving CD29 upregulation to promote cellular adhesion and accelerate the MASH‐to‐HCC transition via integrin‐mediated signalling cascades.

## 4 Discussion

As a metabolic disease, MASLD is influenced by a variety of factors, including genetics and diet, and affects a large population worldwide [1, 2]. The pathological progression of MASLD evolves from simple steatosis to MASH, which can further deteriorate into cirrhosis and even HCC [3, 4]. Currently, effective therapies for MASLD remain limited; therefore, it is crucial to explore its underlying pathogenic mechanisms and potential therapeutic targets [5, 6]. Previous studies have reported the depletion of CD4^+^ T lymphocytes and the increase of CD8^+^ T lymphocytes in MASLD [8, 9, 10]. The accumulation of fatty acids and mitochondrial damage are major causes of CD4^+^ T cell depletion, while the upregulation of CXCR6 and downregulation of FOXO1 are the main reasons for changes in CD8^+^ T cells [8, 9, 10]. In our study, we also confirmed a significant reduction of CD4^+^ T cells and a marked increase of CD8^+^ T cells in both the peripheral blood and liver of MASLD model mice. These findings suggest that the imbalance between CD4^+^ and CD8^+^ T lymphocytes is associated with immune system activation and lipid accumulation under MASLD conditions [22, 23, 24]: lipid accumulation leads to metabolic reprogramming that benefits tumour cells by accelerating their proliferation and migration.

In response to tumour invasion, the activated immune system increases the number of cytotoxic CD8^+^ T lymphocytes to recognise and kill tumour cells. Our study showed that the increase in cytotoxic CD8^+^ T lymphocytes was primarily observed in the MASH stage, whereas a declining trend was detected in the HCC stage. This indicates that during the intermediate stage of disease progression, large numbers of cytotoxic CD8^+^ T lymphocytes are recruited to the liver to kill tumour cells, but at later stages, excessive consumption of these cells leads to functional decline and exhaustion. Meanwhile, CD4^+^ T cells, which primarily play supportive and regulatory roles, were significantly downregulated, partly due to tumour‐mediated suppression [8, 9, 10].

Our study found that Tregs, a regulatory subpopulation of CD4^+^ T lymphocytes, not only did not decrease with total CD4^+^ T cells but even showed a slight increase. Previous studies have shown that Tregs are upregulated in various cancers such as melanoma, lung cancer, breast cancer and liver cancer, where they contribute to tumour progression and immune evasion [44, 45, 46]. In melanoma, HLA class II‐positive melanoma cells can directly activate Tregs, while antigen‐presenting cells (APCs) can present tumour antigens via HLA class II molecules to indirectly activate Tregs. Activated Tregs then secrete cytokines such as IL‐10 and TGF‐β, suppressing the cytotoxic and helper functions of effector T cells, thereby weakening immune surveillance and allowing tumour cells to escape [44]. In lung cancer, cancer cells can secrete TGF‐β and IL‐10 to induce naïve T cells to differentiate into Tregs, and tumour antigens presented by APCs further enhance Treg activation and accumulation in the tumour microenvironment, where Tregs inhibit NK cell‐mediated cytotoxicity [45]. In breast cancer, tumour‐derived chemokines such as CCL22 and CCL1 bind to CCR4 and CCR8 on Tregs, guiding their migration into tumour tissues. Tregs also promote angiogenesis by secreting factors like VEGF, providing nutrients and oxygen for tumour growth and creating pathways for tumour metastasis [46]. Although we did not observe a gradual increase of Tregs within the liver during MASLD progression, we did find a marked stepwise increase in peripheral blood.

To further investigate this phenomenon, we used unsupervised clustering and identified two Treg subpopulations, defined as Treg A and Treg B. Treg B gradually increased with MASLD progression and showed high expression of CD29, which was also identified as a high‐risk scoring gene. Given the pathogenic potential exhibited by the Treg B subpopulation, we redefined it as the CD29‐high Treg subset. These results indicate that due to the high heterogeneity of Tregs, not all Tregs are pathogenic; only certain subpopulations may promote tumour proliferation and migration through specific mechanisms [47, 48].

It is known that multiple Treg subsets are significantly upregulated and promote the progression of HCC. For example, CD39^+^ Tregs can mediate nucleotide metabolic pathways to inhibit the function of CD8^+^ T cells; Nrp1^+^ Tregs can enhance Treg migration ability; CCR4^+^ Tregs can secrete inflammatory factors to strengthen the immunosuppressive function of Tregs [49, 50, 51]. However, previous studies have not reported the mechanism by which Tregs exert pathogenicity through integrin‐mediated adhesion. Through further research on the CD29‐high Treg subpopulation, we hypothesise that CD29's strong adhesion properties enhance Treg adhesion, facilitating their migration. This unique pathological specificity may explain why liver‐infiltrating Tregs did not show a proportional increase in MASLD progression, whereas peripheral Tregs increased dramatically, suggesting enhanced peripheral migration and dissemination. This expansion of pathogenic Treg distribution may ultimately accelerate the transition from MASH to HCC. EMT (epithelial‐mesenchymal transition) is a biological process in which epithelial cells lose polarity and adhesion, acquiring migratory mesenchymal features. EMT is critical in embryonic development, tissue repair, and tumour metastasis, influencing immune cell function within the tumour microenvironment and providing new insights into cancer therapy [52, 53, 54].

As a member of the integrin family, CD29 plays a crucial role in promoting cell proliferation, adhesion and migration [17, 18]. Previous studies have reported that CD29‐enriched extracellular vesicles mediate monocyte migration to endothelial cells and promote liver inflammation [19]. Our study presents a novel perspective by investigating the interaction between immune cells and integrins in MASLD. We demonstrated that Tregs enhance their binding with CD29 under MASLD conditions, and CD29, in turn, enhances Treg‐mediated tumour cell adhesion and EMT induction, driving the malignant progression from MASH to HCC.

While EMT's role in tumour development is well established [52, 53, 54], reports of immune cell‐mediated EMT induction are relatively rare [55, 56]. CD29 has been implicated in EMT‐mediated tumour progression in various cancers: ALDH3B2 promotes cholangio‐carcinoma development through CD29 regulation [57]; organoid coculture and transfer learning experiments showed that CAFs interact with epithelial cells via CD29 and VEGFA‐NRP1 to induce inflammation and EMT pathways, promoting pancreatic ductal adenocarcinoma [58]; EMT‐UC cell populations are associated with chemotherapy resistance and relapse in bladder cancer through COL4A1‐CD29 interactions [59]; in colorectal cancer, THBS2^+^ CAFs are linked to oxaliplatin resistance via COL8A1‐CD29‐mediated EMT activation [60].

CD29‐high Tregs also promote cancer progression through EMT: CD29^+^ Tregs, characterised by high CD29 expression, proliferate robustly in the liver, fill Treg niches and exhibit strong immunosuppressive functions, impairing ICI (immune checkpoint inhibitor) therapy efficacy against liver tumours [61]. Tregs can mediate EMT through the TGF‐β1/Smad pathway, promoting radiation‐induced lung injury (RILI) [62]. Several studies have also linked Tregs to tumour cells via EMT mechanisms: differential expression of resting and effector Tregs is associated with patient survival and key cancer pathways including EMT [63]; after incomplete microwave ablation (iMWA) in HCC mice, residual tumours exhibited TGF‐β activation, increased EMT, Treg infiltration and tumour growth [64]; Treg‐associated prognostic genes are highly expressed in clear cell renal cell carcinoma (ccRCC), promoting tumour proliferation, invasion and Treg infiltration [65]; scRNA‐seq and spatial transcriptomic analyses of CRC tissues at different stages revealed that Tregs interact with EMT‐associated invasive genes during tumour invasion [66].

These studies highlight CD29's crucial role in the tumour microenvironment, suggesting its dual high expression in tumour and immune cells, providing a theoretical foundation for our study's mechanism by which Tregs upregulate CD29 to induce EMT and drive MASLD malignancy. Through MASLD dynamic models and liver single‐cell sequencing, we identified the CD29‐high Treg subpopulation. High CD29 expression was also validated in liver cancer cells using TCGA and GSE76427 datasets. Knocking down CD29 reduced HCC cell proliferation and migration. Our findings indicate that targeting CD29 suppresses tumour cell adhesion and EMT capabilities.

However, this study also has some limitations. First, validation of CD29 knockdown effects on MASLD relied primarily on bioinformatics analysis and in vitro experiments, lacking in vivo drug intervention studies. Meanwhile, considering that the currently used CD29 inhibitors are mainly anti‐CD29 antibodies, which have been applied in preclinical trials but lack clinical trial testing, the applicable scope and efficacy of CD29 inhibitors still need further verification [19]. Second, integrins are also expressed in normal tissues, where they help promote angiogenesis and improve cardiac function. Knocking down CD29 may cause side effects such as bleeding or delayed wound healing [67, 68]. Therefore, it is essential to combine multi‐targeted interventions with novel drug delivery systems to enhance therapeutic efficacy and reduce toxic side effects. Third, although the study focused on CD29 in Tregs, other integrins potentially involved in Treg pathogenicity were not deeply explored.

In conclusion, our study provides new insights into MASLD‐targeted therapy. Our findings elucidate a novel mechanism whereby Tregs enhance pathogenicity via CD29: enhanced Treg‐CD29 interactions in MASLD conditions promote tumour cell adhesion and migration, acting as potential drivers of the transition from MASH to HCC. Targeting CD29 may therefore represent an effective therapeutic strategy to inhibit the malignant progression of MASLD.

## Author Contributions

All authors contributed to the study conception and design. Y.L. contributed mainly to this work and wrote the draft of the paper; Y.L. and M.Y. conceived and designed the study; Y.L., L.L. and M.Y.Z. performed the experiments; Y.L. performed bioinformatics analysis; M.H.Z. and L.J. collected and analysed the data. M.Y. and D.Y. revised the manuscript and edited all drafts of the paper. All authors read and approved the final manuscript.

## Ethics Statement

The study was approved by the Ethics Committee (ECNU‐2023‐9) of Nantong University based on the ethical guidelines of the 1975 Declaration of Helsinki and written informed consent for publication was obtained from all participants. The MASLD model was approved by the Animal Ethics Committee (P20230224‐009) of Nantong University, China.

## Conflicts of Interest

The authors declare no conflicts of interest.

## Supporting information


**Figure S1:** Subpopulation composition of spleen T Cells in MASLD.
**Figure S2:** Functional characteristics of T Cells in MASLD model.
**Figure S3:** Subpopulation of CD8^+^ T Cells in MASLD.
**Figure S4:** Composition and function of spleen Tregs in MASLD.
**Figure S5:** Function characteristics of Tregs in MASLD model.
**Figure S6:** Metabolic activity of Tregs and cell adhesion‐related genes in the pseudotime analysis trajectory of Tregs.
**Table S1:** Clinicopathological features of CD29 expression in LIHC.
**Table S2:** List of primers used in this study.
**Table S3:** List of antibodies used in this study.

## Data Availability

The data that support the findings of this study are available from the corresponding author upon reasonable request.
