# Supplementary material for: Tregs With High CD29 Expression Promote Cell Adhesion and Contribute to the Malignant Transformation of MASLD
Source: Liver Int. 2025 Nov 7;45(12):e70421. doi: 10.1111/liv.70421 (PMC12603612; doi:10.1111/liv.70421)
Supplement: Supplementary file 1 — Figure S1: Subpopulation composition of spleen T Cells in MASLD. Figure S2: Functional characteristics of T Cells in MASLD model. Figure S3: Subpopulation of CD8+ T Cells in MASLD. Figure S4: Composition and function of spleen Tregs in MASLD. Figure S5: Function characteristics of Tregs in MASLD model. Figure S6: Metabolic activity of Tregs and cell adhesion‐related genes in the pseudotime analysis trajectory of Tregs. Table S1: Clinicopathological features of CD29 expression in LIHC. Table S2: List of primers used in this study. Table S3: List of antibodies used in this study. [file LIV-45-0-s001.zip › liv70421-sup-0001-FigureS1-S6-TableS1-S3.docx]

**Tregs with high CD29 expression promote cell adhesion and contribute to the malignant transformation of MASLD**

**Yuming Lu^1^,** **Luyin Liu^1^, Mengya Zhou^1^,** **Minghui Zou^1^, Linling Ju^1^, Dengfu Yao^2*^, Min Yao^1*^**

^1^Department of Immunology, Medical School of Nantong University, Nantong 226001, Jiangsu Province, China

^2^Research Center of Clinical Medicine, Affiliated Hospital of Nantong University, Nantong 226001, Jiangsu Province, China

**Table of Contents**

[**Supplementary Materials and Methods** 2](#_Toc209550278)

[**Supplementary Figures** 8](#_Toc209550279)

[Figure S1 Lu YM, et al 8](#_Toc209550280)

[Figure S2 Lu YM, et al 9](#_Toc209550281)

[Figure S3 Lu YM, et al 10](#_Toc209550282)

[Figure S4 Lu YM, et al 11](#_Toc209550283)

[Figure S5 Lu YM, et al 12](#_Toc209550284)

[Figure S6 Lu YM, et al 14](#_Toc209550285)

[**Supplementary Tables** 16](#_Toc209550286)

[Supplementary Table 1. Clinicopathological features of CD29 expression in LIHC 16](#_Toc209550287)

[Supplementary Table 2. List of primers used in this study 17](#_Toc209550288)

[Supplementary Table 3. List of antibodies used in this study 18](#_Toc209550289)

# **Supplementary Materials and Methods**

**Isolation of peripheral blood mononuclear cells (PBMCs) from mice, as well as nonparenchymal cells (NPCs) and mononuclear cells (MNCs) from the mouse liver**

First, the mice were anesthetized with isoflurane (Sigma Aldrich, USA). Peripheral blood was obtained via retro - orbital bleeding and collected in EDTA - K2 anticoagulant tubes (KWS, China), followed by centrifugation at 3000 rpm for 5 minutes. To isolate mouse PBMCs, diluted whole blood was gently layered over PBMC separation medium and centrifuged at 2000 rpm for 30 minutes. The abdominal cavity was opened, and the liver was perfused with PBS. The liver was then extracted, washed with PBS, and placed in a dish containing type II collagenase (Solarbio, China) for digestion. The liver was minced and placed in a 70 μm filter (Biologix, China), ground with a pestle, and appropriately supplemented with DMEM (Cytiva, China) for filtration. The sample was then subject to centrifugation at 1500 rpm for 5 minutes. The supernatant was discarded, and the pellet was washed with PBS containing 2% fetal bovine serum (Gibco, USA) and centrifuged at 1500 rpm for 5 minutes to obtain NPCs. Percoll (Solarbio, China) was diluted to 40% and 70% concentrations, and NPCs were resuspended in 40% Percoll, gently layered over 70% Percoll and centrifuged at 2000 rpm for 30 minutes to obtain liver MNCs. The isolated NPCs were used for single-cell sequencing, whereas the MNCs were used for flow cytometry.

**Flow Cytometry**

Both mouse PBMCs and liver MNCs were prepared as single-cell suspensions in PBS containing 2% FBS. Surface flow cytometry antibodies were added for staining. The surface antibodies used included those against CD45, CD3, CD4, CD8, and CD25 (BioLegend, USA). Prior to intracellular antibody staining, a fixation and permeabilization solution (BioLegend, USA) was added to fix and permeabilize the cells. Subsequently, intracellular staining was performed using the nuclear flow cytometry antibody Foxp3 (BioLegend, USA). The surface and intracellular flow cytometry antibodies used are listed in Table S3.

**Determination of Serum Biochemical Parameters**

Mouse serum was analyzed via test kits (Njjcbio, China) for alanine aminotransferase (ALT), aspartate aminotransferase (AST), glucose (GLU), low-density lipoprotein (LDL), triglyceride (TG), and total cholesterol (TC) levels.

**Histopathology**

For paraffin section preparation, mouse liver tissue was fixed in 4% paraformaldehyde (Biosharp, China) for 24 hours, embedded in paraffin, and sectioned to a thickness of 4 μm. The sections were stained with hematoxylin and eosin (H&E) and imaged using a Leica microscope (Germany). For cryosection preparation, mouse liver tissue was immediately frozen in liquid nitrogen and embedded. Using a cryostat, 8–10 μm thick cryosections were prepared. After thawing for half an hour, the sections were stained with an Oil Red O staining kit (Solarbio, China) and imaged via a Leica microscope (Germany). The obtained images were then subjected to histopathological analysis.

**Immunohistochemistry (IHC)**

Mouse liver tissue was fixed in 4% paraformaldehyde (Biosharp, China) for 24 hours, embedded in paraffin, and sectioned into 4-μm sections. After blocking nonspecific binding sites, primary antibodies against Foxp3, ITGβ1, E-cadherin, N-cadherin, Vimentin, and Snail were incubated with the samples overnight at 4 °C. The next day, the sections were incubated with horseradish peroxidase (HRP) - labeled secondary antibodies at room temperature for 1 hour, followed by H&E staining. Images were captured using a Leica microscope (Germany) and quantitatively analyzed via ImageJ.

**Immunofluorescence (IF)**

Mouse liver tissue was fixed in 4% paraformaldehyde (Biosharp, China) for 24 hours and permeabilized with Triton X-100. Samples were incubated with primary antibodies against Foxp3 and ITGβ1overnight at 4 °C. The next day, the sections were incubated with secondary antibodies at room temperature for 1 hour, followed by a 5-minute incubation with DAPI. Images were acquired via a confocal microscope.

**Cell Proliferation Assay**

Cell proliferation was measured via the Cell Counting Kit - 8 (CCK - 8) (Dojindo, Japan). On Day 1, the transfected HepG2 and LM3 cells were seeded in a 96 - well plate at densities of 2000 cells per well and 1000 cells per well, respectively, and incubated overnight in a CO₂ incubator. On Day 2, the prepared CCK - 8 working solution was added to each well and incubated at 37 °C for 2 hours. After incubation, absorbance was measured at 450 nm via a microplate reader. The absorbance was then measured again at 24 h, 48 h, 72 h, and 96 h.

**Cell Invasion and Migration Assay**

A 24well plate (Corning, USA) was used as the culture container to assess cell invasion and migration capabilities. For the invasion assay, the upper chamber was precoated in Matrigel (Corning, USA) and incubated in a CO₂ incubator for 30 minutes. HepG2 and LM3 cells transfected with either the control vector or ITGβ1-siRNA for 48 hours were collected and added to the upper chamber. The lower chamber was filled with DMEM containing 20% fetal bovine serum. For the migration assay, HepG2 and LM3 cells transfected with either the control vector or ITGβ1-siRNA for 48 hours were directly added to the upper chamber. The lower chamber was also filled with DMEM containing 20% fetal bovine serum. After incubation for 72 hours, the cells were fixed with 4% paraformaldehyde for 10 minutes and stained with crystal violet solution (Beyotime, China) for 10 minutes. The dye was gently removed from the upper chamber using a cotton swab. Images were captured via a Leica microscope, and the number of invading and migrating cells was quantified using ImageJ.

**RT**‒**qPCR**

After HepG2 and LM3 cells were transfected with ITGβ1-siRNA or the control vector (GenePharma, China) for 48 hours, the cells were harvested. RNA was extracted from the cells via TRIzol reagent (Ambion). Then, the extracted RNA was reverse transcribed into cDNA using a reverse transcription kit (Vazyme, China). Quantitative RT‒PCR analysis was performed using ChamQ Blue Universal SYBR qPCR Master Mix (Vazyme, China) and a LightCycler 96 detection system (Roche, Switzerland). Here, β-actin was used as an internal control, and gene mRNA expression levels were analyzed via the 2 - ΔΔCt method. The sequences of primers used are listed in Table S2.

**Western blot**

After HepG2 and LM3 cells were transfected with ITGβ1-siRNA or the vector control (GenePharma, China) for 48 hours, the cells were harvested. On ice, protease and phosphatase inhibitors were mixed with RIPA lysis buffer (Epizyme, China) at a 1:100 ratio to extract cellular proteins. The protein concentration was determined using the BCA assay kit (Epizyme, China). The 5X loading buffer (Solarbio, China) was mixed with the protein - containing RIPA lysis buffer at a 1:4 ratio, and the mixture was boiled for 10 minutes before being loaded onto a 4% to 15% SDS‒PAGE gel (Epizyme, China) for electrophoresis. Proteins were then transferred onto a 200 μm nitrocellulose membrane (NC membrane, Cytiva, China) via a semidry transfer apparatus (Bio Rad, USA). The membrane was blocked with 5% skim milk (Biosharp, China) at room temperature for 2 hours, followed by incubation with appropriately diluted primary antibodies overnight at 4 °C. The next day, after washing with TBST, the membrane was incubated with the corresponding secondary antibodies at room temperature for 2 hours. After washing with TBST again, the membranes were imaged via a gel imaging system (Tanon, China), and quantitative analysis was performed via Image J. The details of the primary and secondary antibodies used are listed in Table S3.

**Single-Cell Sequencing Technology**

Fresh nonparenchymal cells (NPCs) derived from the livers of 26-week-old mice in the three sample groups were prepared as a single-cell suspensions. The prepared single-cell suspensions were processed for single-cell RNA sequencing (scRNA - seq) via the 10 × Genomics platform (Gene Denovo). Cell Ranger version 6.1.0, Seurat version 4.1.0, and CellChat version 2.1.0 were utilized for the analysis.

**Bioinformatics Analysis**

R version 4.1.3 was used for the analysis. Transcriptome sequencing data from 529 liver cancer patients were downloaded from TCGA database, and data from 167 patients were obtained from the GSE76427 database. TCGA database includeds 369 tumor samples and 160 normal samples, whereas the GSE76427 database includeds 115 tumor samples and 52 normal samples. The Wilcoxon rank-sum test was applied to compare the differences in ITGβ1 mRNA expression between LIHC patients. To assess the prognostic significance of ITGβ1 in LIHC, samples with incomplete clinical information were excluded, and 297 samples from the TCGA database and 95 samples from the GSE76427 database with complete clinical data were retained. Survival analysis was performed via the Kaplan–Meier method and log-rank test. Additionally, Cox univariate and multivariate regression models were constructed to estimate the risk score of clinical relevance and the ITGβ1 gene in LIHC patients from the TCGA database. Furthermore, Pearson correlation analysis was conducted to determine the correlation between Foxp3 and ITGβ1 expression in LIHC.

**Statistical Analysis**

All the statistical analyses were performed via GraphPad Prism 8.0 and SPSS software. Student's t - test, one - way ANOVA, and two - way ANOVA were used to assess differences between groups. All experiments were independently repeated at least three times. The data are presented as the means ± standard deviation (mean ± SD), and a p-value < 0.05 was considered statistically significant.

# **Supplementary Figures**

## **Figure S1 Lu YM, et al**


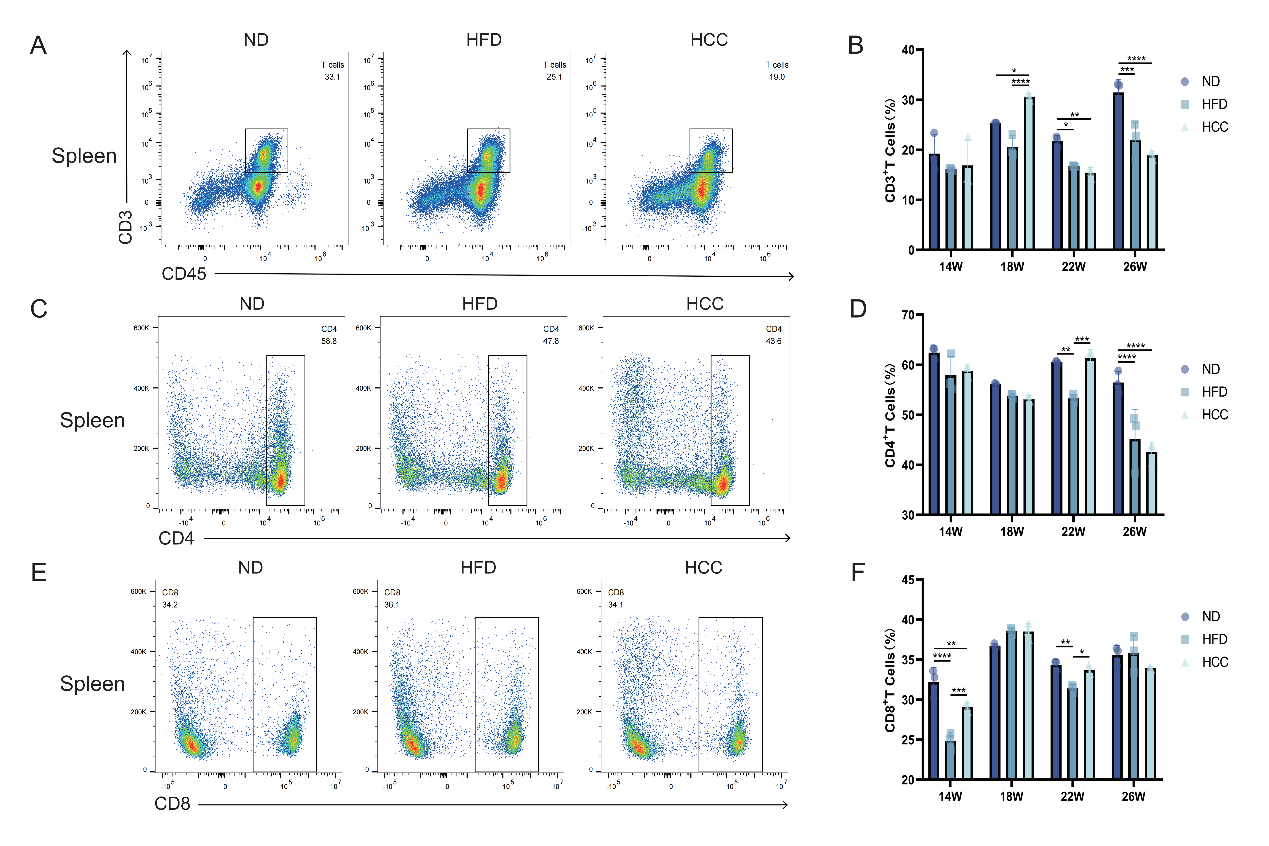


**Supplementary Figure 1 Subpopulation Composition of Spleen T Cells in MASLD.**

(**A-B**) Flow cytometry analysis of CD3^+^ T lymphocytes proportions in the spleen at different stages in ND, HFD, and HCC mice (n = 3/group). (**C-D**) Flow cytometry analysis of spleen CD4^+^ T cells ratio at different stages in ND, HFD, and HCC mice (n = 3/group). (**E-F**) Flow cytometry analysis of CD8^+^ T lymphocytes proportions in the spleen at different stages in ND, HFD, and HCC mice (n = 3/group). **ND**: normal diet, **HFD**: high-fat diet, **HCC**: HFD + carcinogen. ^*^*p* < 0.05, ^**^*p* < 0.01, ^***^*p* < 0.001, ^****^*p* < 0.0001.

## **Figure S2 Lu YM, et al**


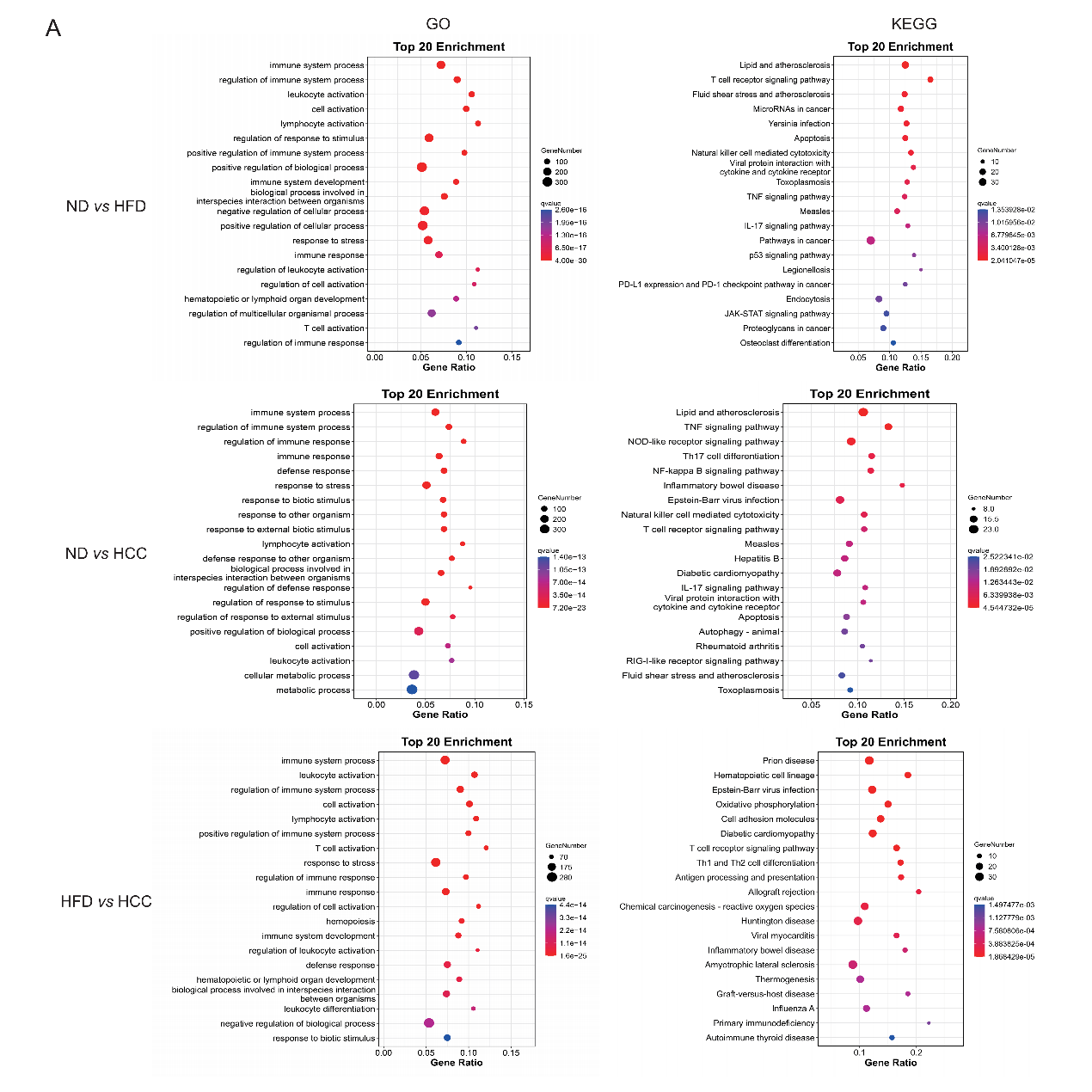


**Supplementary Figure 2 Functional Characteristics of T Cells in MASLD Model.**

(**A**) GO and KEGG enrichment analyses of hepatic T lymphocytes from the ND, HFD, and HCC groups. **ND**: normal diet, **HFD**: high fat diet, **HCC**: HFD + carcinogen.

## **Figure S3 Lu YM, et al**


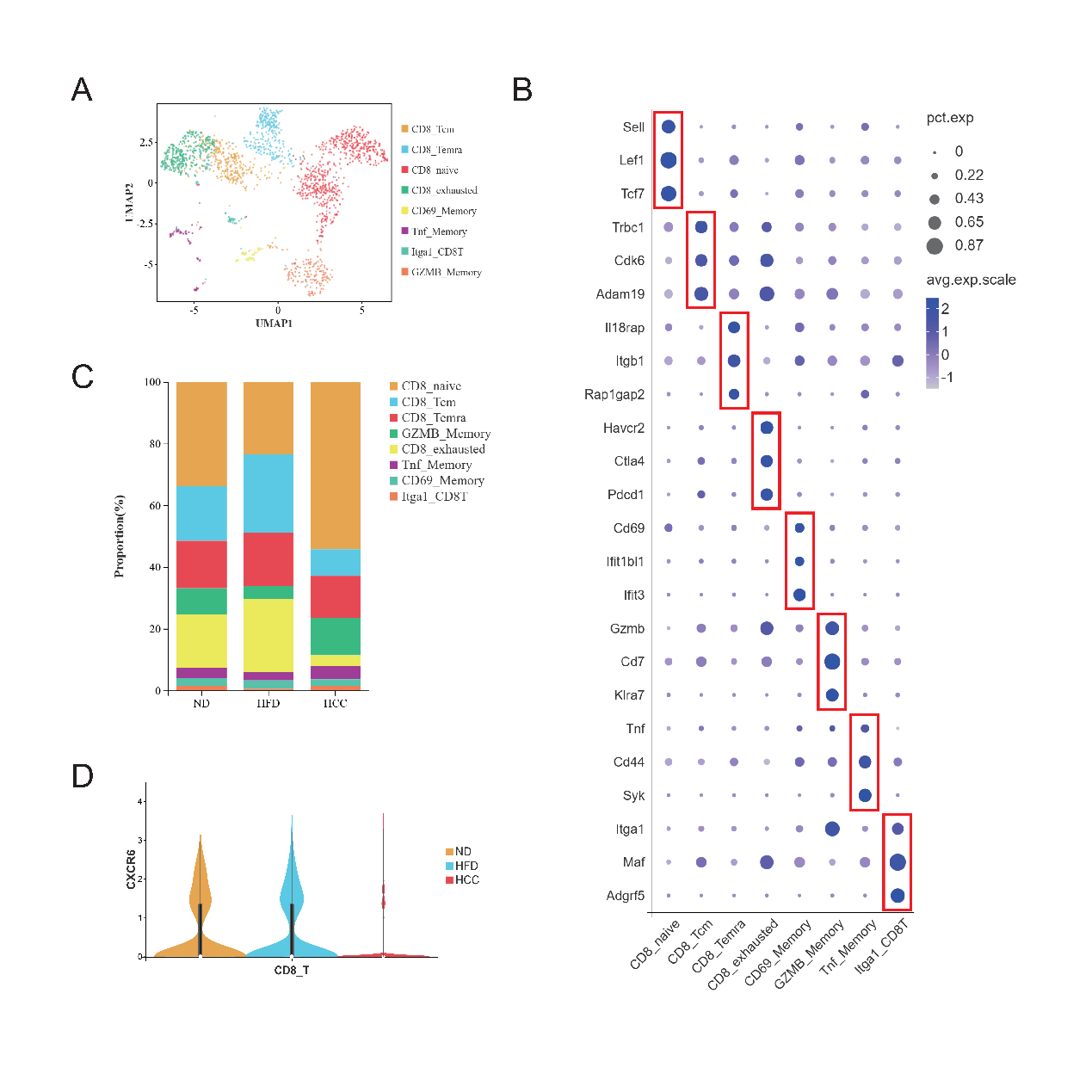


**Supplementary Figure 3 Subpopulation of CD8^+^ T Cells in MASLD.**

(**A**) UMAP plot of CD8^+^ T lymphocytes from MASLD model mice, categorized by cell type and sample group. (**B**) Bubble plot showing marker genes for CD8^+^ T lymphocyte subpopulations, with three marker genes for each subpopulation. (**C**) Proportions of CD8^+^ T lymphocyte subpopulations in the ND, HFD and HCC groups. (**D**) Violin plots of CXCR6^+^ CD8^+^ T lymphocytes. **ND**: normal diet; **HFD**: high-fat diet; **HCC**: HFD + carcinogen.

## **Figure S4 Lu YM, et al**

**
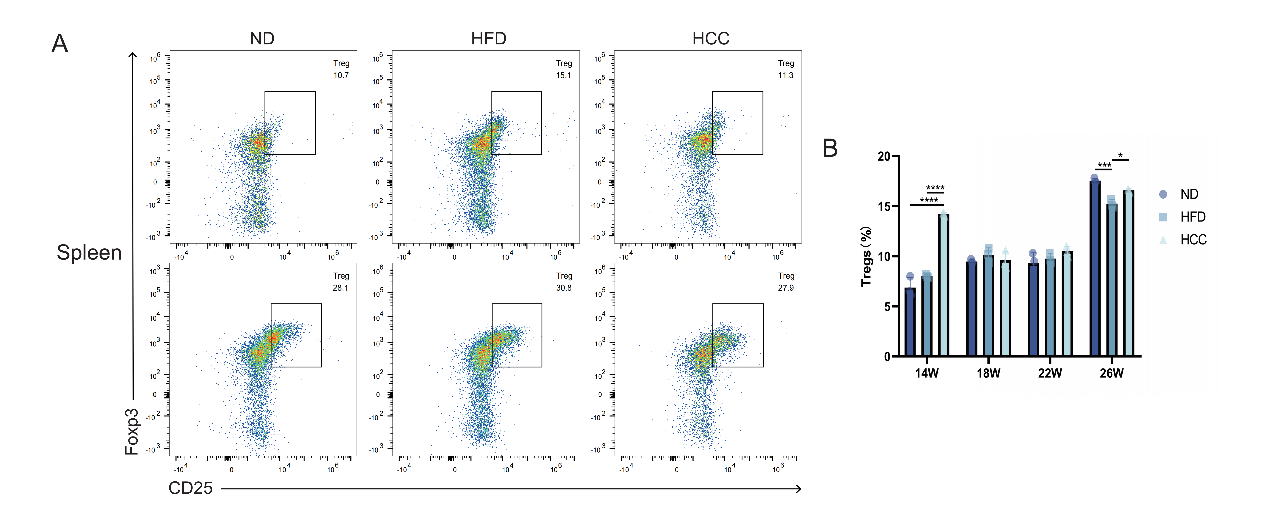
**

**Supplementary Figure 4 Composition and Function of Spleen Tregs in MASLD.**

(**A-B**) Flow cytometry analysis of Tregs proportions in the spleen at different stages in ND, HFD, and HCC mice (n = 3/group). **ND**: normal diet, **HFD**: high-fat diet, **HCC**: HFD + carcinogen. ^*^*p* < 0.05, ^**^*p* < 0.01, ^***^*p* < 0.001, ^****^*p* < 0.0001.

## **Figure S5 Lu YM, et al**

**
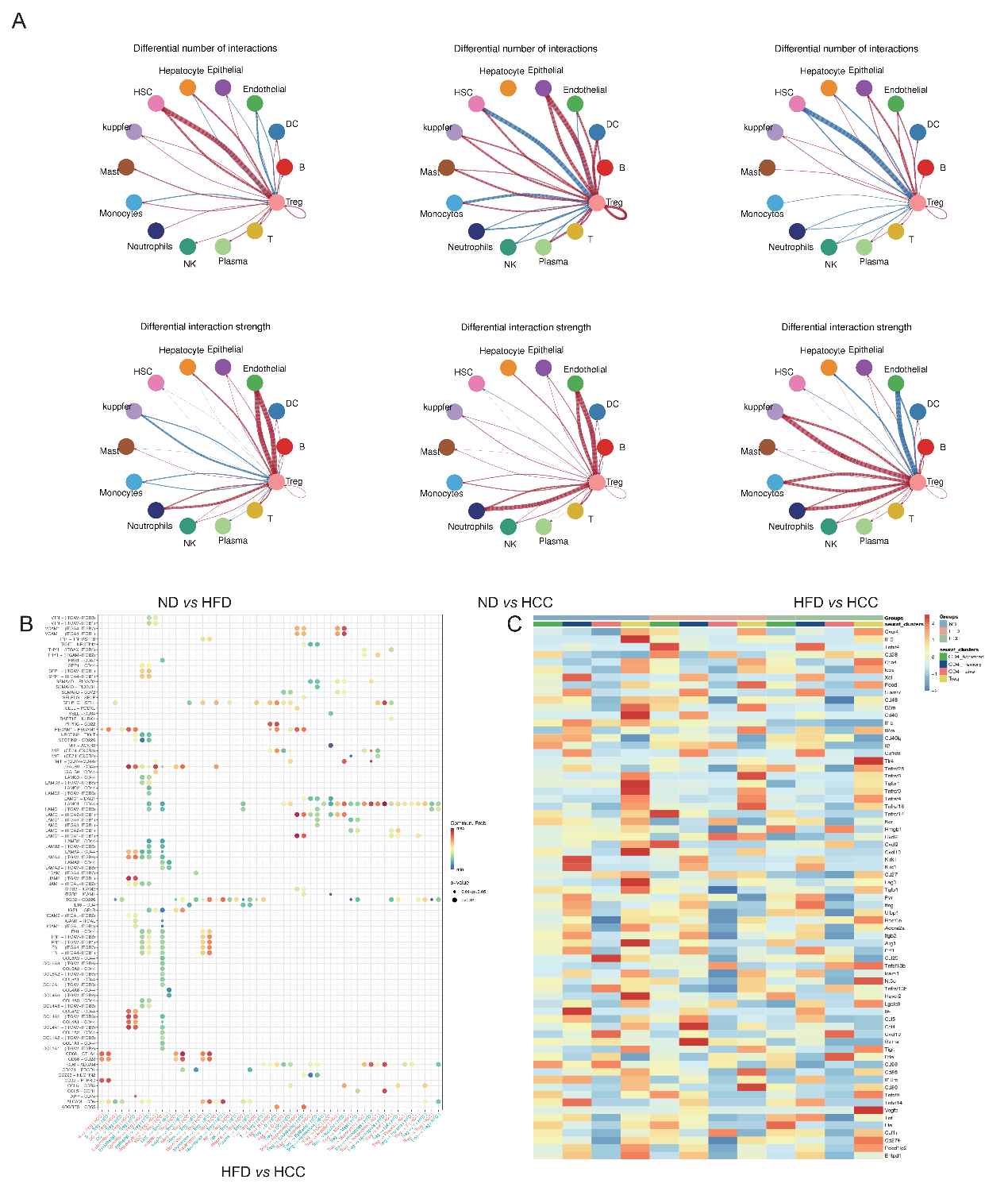
**

**Supplementary Figure 5 Function Characteristics of Tregs in MASLD Model.**

(**A**) Number and intensity of cellular communication between Tregs and other cells in the ND, HFD, and HCC groups. (**B**) Bubble plot of significant ligand‒receptor interactions between Tregs and other cells in the HFD and HCC groups. (**C**) Heatmap of cytokine expression by CD4^+^ T lymphocyte subpopulations in the ND, HFD, and HCC groups. **ND**: normal diet; **HFD**: high-fat diet; **HCC**: HFD + carcinogen.

## **Figure S6 Lu YM, et al**

**
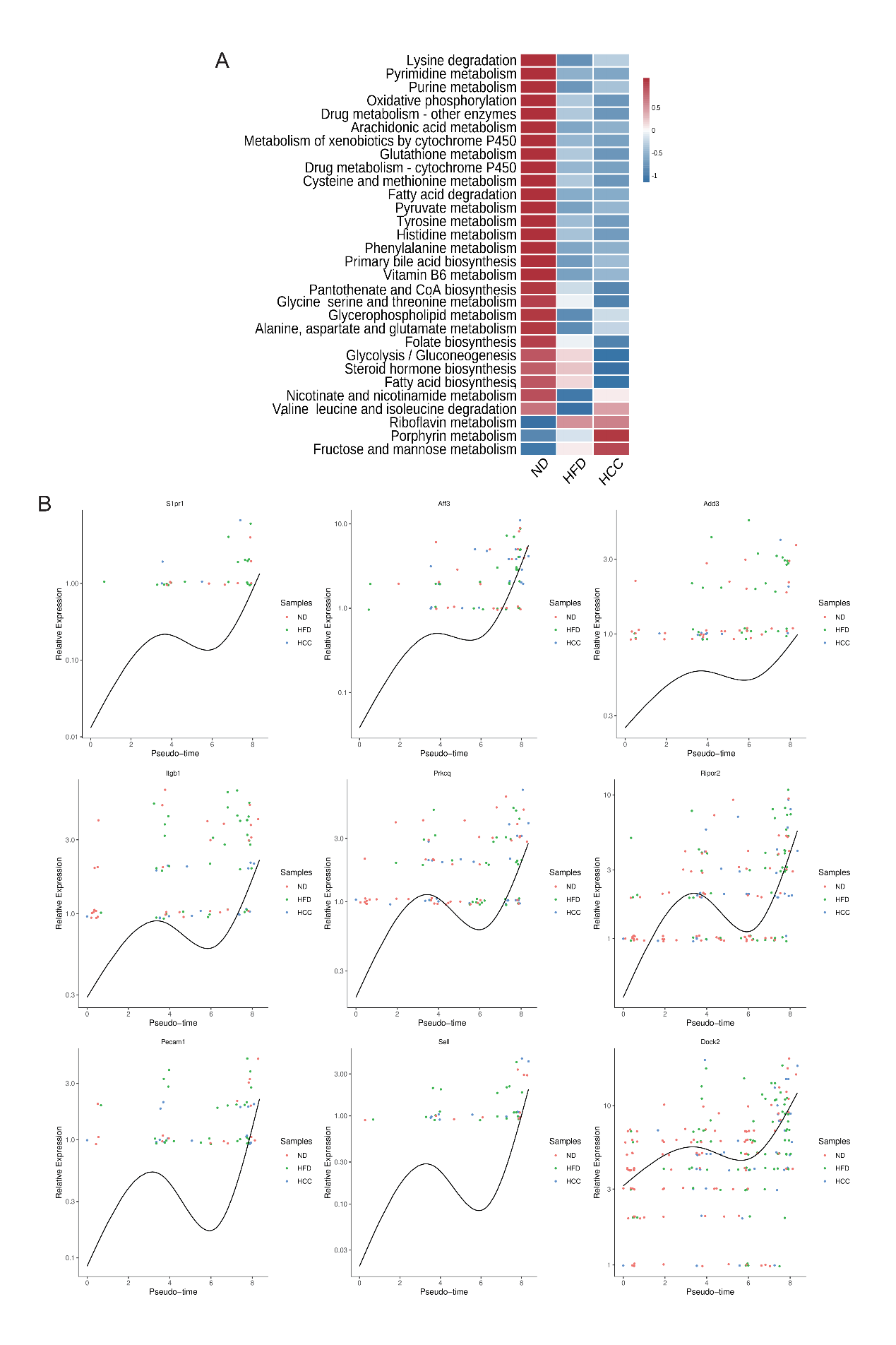
**

**Supplementary Figure 6 Metabolic Activity of Tregs and Cell Adhesion-related Genes in the Pseudotime Analysis Trajectory of Tregs.**

(**A**) Quantification of the metabolic activity of Tregs in the livers of ND, HFD, and HCC mice using the Metabolism package. (**B**) Cell adhesion-related genes in the pseudotime analysis trajectory of Tregs. **ND**: normal diet, **HFD**: high-fat diet, **HCC**: HFD + carcinogen.

# **Supplementary Tables**

## **Supplementary Table 1. Clinicopathological features of CD29 expression in LIHC**

| Group | CD29 expression (n=220) | | | *P* value |
| --- | --- | --- | --- | --- |
|  | Total Low High | | |  |
| **CD29**  **Age (years)**  ≤60  >60  **Gender**  Male  Female  **Tumor**  T1-2  T3-4  **Node**  N0  N1-2  [**Metastasis**](https://baike.baidu.com/item/metastasis/53056350?fromModule=lemma_inlink)  M0  M1  **Stage**  Stage I-II  Stage III-IV  **Grade**  Grade I-II  Grade III-IV | 220  123  97  151  69  155  65  217  3  217  3  153  67  123  97 | 101 (45.91%)  51  50  74  27  79  22  101  0  99  2  79  22  62  39 | 119 (54.09%)  72  47  77  42  76  43  116  3  118  1  74  45  61  58 | 0.0273^*^ |
|  |  |  |  | 0.1109 |
|  |  |  |  | 0.0034^**^ |
|  |  |  |  | 0.0818 |
|  |  |  |  | 0.5631 |
|  |  |  |  | 0.0052^**^ |
|  |  |  |  | 0.0869 |
|  |  |  |  |  |

p < 0.05, ** p < 0.01

## **Supplementary Table 2. List of primers used in this study**

| Gene | Sequences (5’ to 3’) | Application |
| --- | --- | --- |
| ITGB1  Foxp3  E-Cadherin  N-Cadherin  Vimentin  Snail  Pecam1  Jam2 | Forward:CTGTGATGCCTTACATTAGCAC  Reverse:ATCCAAATTTCCAGATATGCGC  Forward:CTCTTCTTCCTTGAACCCCAT  Reverse:CTGGAGGAGTGCCTGTAAG  Forward:AGTCACTGACACCAACGATAAT  Reverse:ATCGTTGTTCACTGGATTTGTG  Forward:CGATAAGGATCAACCCCATACA  Reverse:TTCAAAGTCGATTGGTTTGACC  Forward:TACCAAGACCTGCTCAATGTTAAGATG  Reverse:AATCCTGCTCTCCTCGCCTTC  Forward:TCAGATGAGGACAGTGGGAAAGG  Reverse:AAGGAAGAGACTGAAGTAGAGGAGAAG  Forward:TCGTGGTCAACATAACAGAACT  Reverse:TTGAGTCTGTGACACAATCGTA  Forward:CAATTCTGTTGGATATCGCAGG  Reverse:CCTTTCCTCTGAGCATAGCATA | qPCR  qPCR  qPCR  qPCR  qPCR  qPCR  qPCR  qPCR |

## **Supplementary Table 3. List of antibodies used in this study**

| **Antibodies** | **Source** | **Identifier** |
| --- | --- | --- |
| Beta Actin Monoclonal antibody | Proteintech | 66009-1-Ig |
| Integrin Beta 1 Monoclonal antibody | Proteintech | 66315-1-Ig |
| FOXP3 Polyclonal antibody | Proteintech | 22228-1-AP |
| E-cadherin Monoclonal antibody | Proteintech | 60335-1-Ig |
| N-cadherin Monoclonal antibody | Proteintech | 66219-1-Ig |
| Vimentin Monoclonal antibody | Proteintech | 60330-1-Ig |
| SNAI1 Polyclonal antibody | Proteintech | 13099-1-AP |
| CD31 Monoclonal antibody | Proteintech | 66065-2-Ig |
| JAM2 Polyclonal antibody | Proteintech | 12972-1-AP |
| HRP, Goat Anti-Mouse IgG | Abbkine | A21010 |
| HRP, Goat Anti-Rabbit IgG | Abbkine | A21020 |
| PerCP/Cyanine5.5 anti-mouse CD45 Antibody | Biolegend | 103132 |
| FITC anti-mouse CD3 Antibody | Biolegend | 100203 |
| APC/Cyanine7 anti-mouse CD4 Antibody | Biolegend | 100414 |
| PE/Cyanine7 anti-mouse CD8a Antibody | Biolegend | 100722 |
| APC anti-mouse CD25 Antibody | Biolegend | 102012 |
| PE anti-mouse FOXP3 Antibody | Biolegend | 126404 |
| APC Rat IgG1, λ Isotype Ctrl Antibody | Biolegend | 401904 |
| PE Rat IgG2b, κ Isotype Ctrl Antibody | Biolegend | 400608 |
