# Supplementary material for: Tregs With High CD29 Expression Promote Cell Adhesion and Contribute to the Malignant Transformation of MASLD
Source: Liver Int. 2025 Nov 7;45(12):e70421. doi: 10.1111/liv.70421 (PMC12603612; doi:10.1111/liv.70421)
Supplement: Supplementary file 1 — Figure S1: Subpopulation composition of spleen T Cells in MASLD. Figure S2: Functional characteristics of T Cells in MASLD model. Figure S3: Subpopulation of CD8+ T Cells in MASLD. Figure S4: Composition and function of spleen Tregs in MASLD. Figure S5: Function characteristics of Tregs in MASLD model. Figure S6: Metabolic activity of Tregs and cell adhesion‐related genes in the pseudotime analysis trajectory of Tregs. Table S1: Clinicopathological features of CD29 expression in LIHC. Table S2: List of primers used in this study. Table S3: List of antibodies used in this study. [file LIV-45-0-s001.zip › liv70421-sup-0008-TableS1@Table S1.docx]

**Supplementary Table 1. Clinicopathological features of CD29 expression in LIHC**

| Group | CD29 expression (n=220) | | | *P* value |
| --- | --- | --- | --- | --- |
|  | Total Low High | | |  |
| **CD29**  **Age (years)**  ≤60  >60  **Gender**  Male  Female  **Tumor**  T1-2  T3-4  **Node**  N0  N1-2  [**Metastasis**](https://baike.baidu.com/item/metastasis/53056350?fromModule=lemma_inlink)  M0  M1  **Stage**  Stage I-II  Stage III-IV  **Grade**  Grade I-II  Grade III-IV | 220  123  97  151  69  155  65  217  3  217  3  153  67  123  97 | 101 (45.91%)  51  50  74  27  79  22  101  0  99  2  79  22  62  39 | 119 (54.09%)  72  47  77  42  76  43  116  3  118  1  74  45  61  58 | 0.0273^*^ |
|  |  |  |  | 0.1109 |
|  |  |  |  | 0.0034^**^ |
|  |  |  |  | 0.0818 |
|  |  |  |  | 0.5631 |
|  |  |  |  | 0.0052^**^ |
|  |  |  |  | 0.0869 |
|  |  |  |  |  |

p < 0.05, ** p < 0.01
