# Supplementary material for: Tregs With High CD29 Expression Promote Cell Adhesion and Contribute to the Malignant Transformation of MASLD
Source: Liver Int. 2025 Nov 7;45(12):e70421. doi: 10.1111/liv.70421 (PMC12603612; doi:10.1111/liv.70421)
Supplement: Supplementary file 1 — Figure S1: Subpopulation composition of spleen T Cells in MASLD. Figure S2: Functional characteristics of T Cells in MASLD model. Figure S3: Subpopulation of CD8+ T Cells in MASLD. Figure S4: Composition and function of spleen Tregs in MASLD. Figure S5: Function characteristics of Tregs in MASLD model. Figure S6: Metabolic activity of Tregs and cell adhesion‐related genes in the pseudotime analysis trajectory of Tregs. Table S1: Clinicopathological features of CD29 expression in LIHC. Table S2: List of primers used in this study. Table S3: List of antibodies used in this study. [file LIV-45-0-s001.zip › liv70421-sup-0009-TableS2@Table S2.docx]

**Supplementary Table 2. List of primers used in this study**

| Gene | Sequences (5’ to 3’) | Application |
| --- | --- | --- |
| ITGB1  Foxp3  E-Cadherin  N-Cadherin  Vimentin  Snail  Pecam1  Jam2 | Forward:CTGTGATGCCTTACATTAGCAC  Reverse:ATCCAAATTTCCAGATATGCGC  Forward:CTCTTCTTCCTTGAACCCCAT  Reverse:CTGGAGGAGTGCCTGTAAG  Forward:AGTCACTGACACCAACGATAAT  Reverse:ATCGTTGTTCACTGGATTTGTG  Forward:CGATAAGGATCAACCCCATACA  Reverse:TTCAAAGTCGATTGGTTTGACC  Forward:TACCAAGACCTGCTCAATGTTAAGATG  Reverse:AATCCTGCTCTCCTCGCCTTC  Forward:TCAGATGAGGACAGTGGGAAAGG  Reverse:AAGGAAGAGACTGAAGTAGAGGAGAAG  Forward:TCGTGGTCAACATAACAGAACT  Reverse:TTGAGTCTGTGACACAATCGTA  Forward:CAATTCTGTTGGATATCGCAGG  Reverse:CCTTTCCTCTGAGCATAGCATA | qPCR  qPCR  qPCR  qPCR  qPCR  qPCR  qPCR  qPCR |
