# Supplementary material for: Tregs With High CD29 Expression Promote Cell Adhesion and Contribute to the Malignant Transformation of MASLD
Source: Liver Int. 2025 Nov 7;45(12):e70421. doi: 10.1111/liv.70421 (PMC12603612; doi:10.1111/liv.70421)
Supplement: Supplementary file 1 — Figure S1: Subpopulation composition of spleen T Cells in MASLD. Figure S2: Functional characteristics of T Cells in MASLD model. Figure S3: Subpopulation of CD8+ T Cells in MASLD. Figure S4: Composition and function of spleen Tregs in MASLD. Figure S5: Function characteristics of Tregs in MASLD model. Figure S6: Metabolic activity of Tregs and cell adhesion‐related genes in the pseudotime analysis trajectory of Tregs. Table S1: Clinicopathological features of CD29 expression in LIHC. Table S2: List of primers used in this study. Table S3: List of antibodies used in this study. [file LIV-45-0-s001.zip › liv70421-sup-0010-TableS3@Table S3.docx]

**Supplementary Table 3. List of antibodies used in this study**

| **Antibodies** | **Source** | **Identifier** |
| --- | --- | --- |
| Beta Actin Monoclonal antibody | Proteintech | 66009-1-Ig |
| Integrin Beta 1 Monoclonal antibody | Proteintech | 66315-1-Ig |
| FOXP3 Polyclonal antibody | Proteintech | 22228-1-AP |
| E-cadherin Monoclonal antibody | Proteintech | 60335-1-Ig |
| N-cadherin Monoclonal antibody | Proteintech | 66219-1-Ig |
| Vimentin Monoclonal antibody | Proteintech | 60330-1-Ig |
| SNAI1 Polyclonal antibody | Proteintech | 13099-1-AP |
| CD31 Monoclonal antibody | Proteintech | 66065-2-Ig |
| JAM2 Polyclonal antibody | Proteintech | 12972-1-AP |
| HRP, Goat Anti-Mouse IgG | Abbkine | A21010 |
| HRP, Goat Anti-Rabbit IgG | Abbkine | A21020 |
| PerCP/Cyanine5.5 anti-mouse CD45 Antibody | Biolegend | 103132 |
| FITC anti-mouse CD3 Antibody | Biolegend | 100203 |
| APC/Cyanine7 anti-mouse CD4 Antibody | Biolegend | 100414 |
| PE/Cyanine7 anti-mouse CD8a Antibody | Biolegend | 100722 |
| APC anti-mouse CD25 Antibody | Biolegend | 102012 |
| PE anti-mouse FOXP3 Antibody | Biolegend | 126404 |
| APC Rat IgG1, λ Isotype Ctrl Antibody | Biolegend | 401904 |
| PE Rat IgG2b, κ Isotype Ctrl Antibody | Biolegend | 400608 |
