# Supplementary figures and images for: Tregs With High CD29 Expression Promote Cell Adhesion and Contribute to the Malignant Transformation of MASLD
Source: Liver Int. 2025 Nov 7;45(12):e70421. doi: 10.1111/liv.70421 (PMC12603612; doi:10.1111/liv.70421)

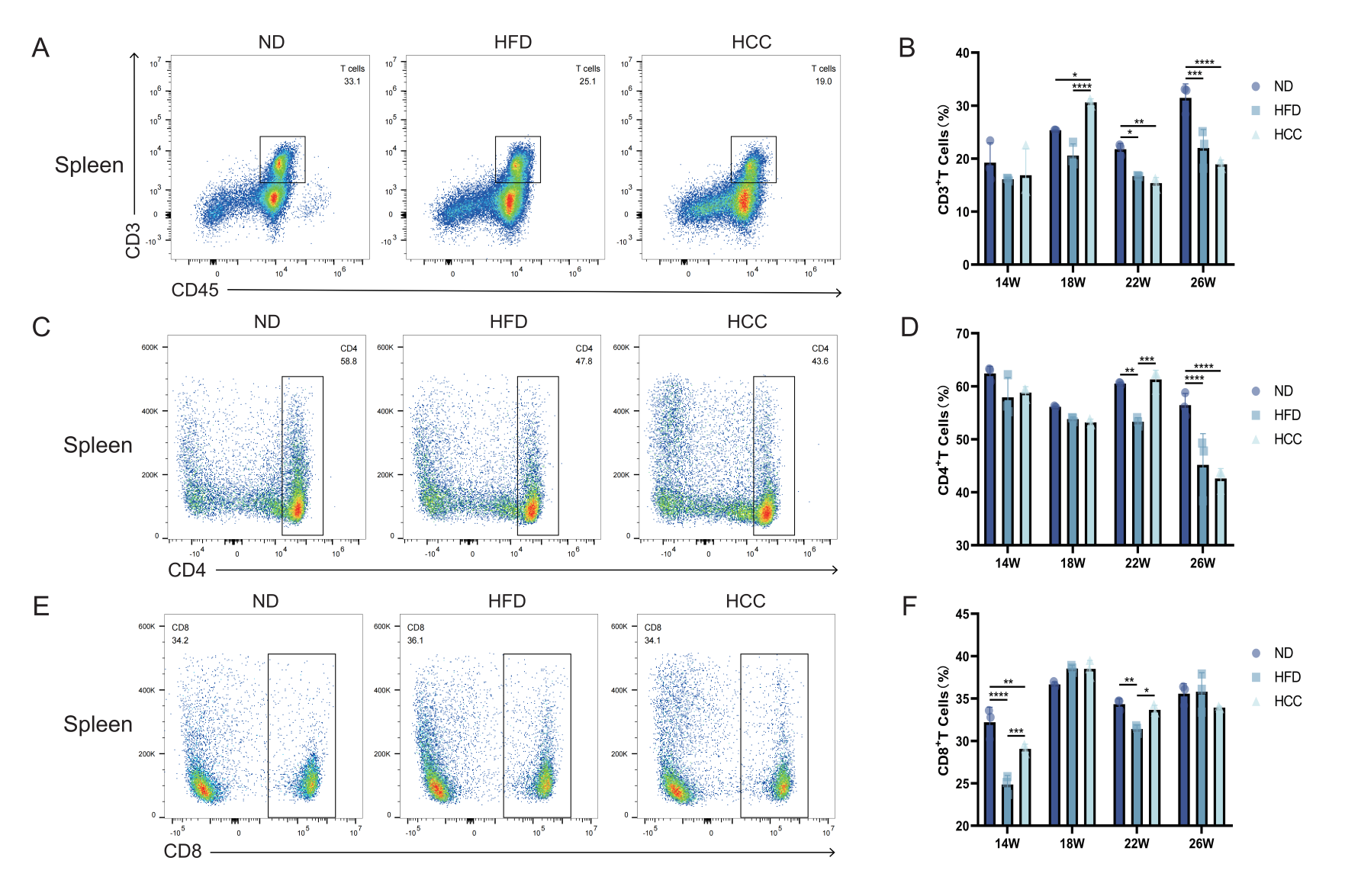

Supplement: Supplementary file 1 — Figure S1: Subpopulation composition of spleen T Cells in MASLD. Figure S2: Functional characteristics of T Cells in MASLD model. Figure S3: Subpopulation of CD8+ T Cells in MASLD. Figure S4: Composition and function of spleen Tregs in MASLD. Figure S5: Function characteristics of Tregs in MASLD model. Figure S6: Metabolic activity of Tregs and cell adhesion‐related genes in the pseudotime analysis trajectory of Tregs. Table S1: Clinicopathological features of CD29 expression in LIHC. Table S2: List of primers used in this study. Table S3: List of antibodies used in this study. [file LIV-45-0-s001.zip › liv70421-sup-0002-FigureS1@Figure S1.png]

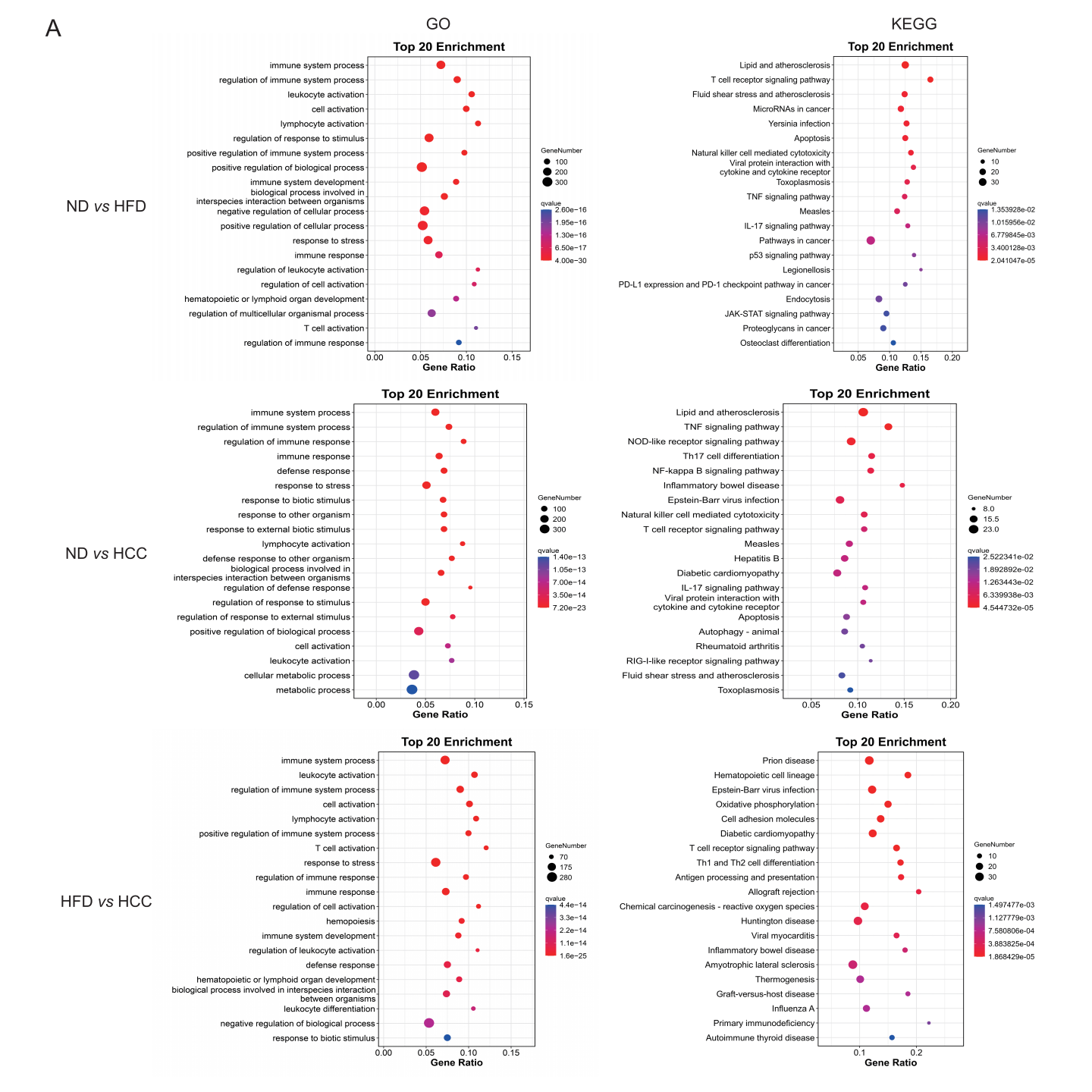

Supplement: Supplementary file 1 — Figure S1: Subpopulation composition of spleen T Cells in MASLD. Figure S2: Functional characteristics of T Cells in MASLD model. Figure S3: Subpopulation of CD8+ T Cells in MASLD. Figure S4: Composition and function of spleen Tregs in MASLD. Figure S5: Function characteristics of Tregs in MASLD model. Figure S6: Metabolic activity of Tregs and cell adhesion‐related genes in the pseudotime analysis trajectory of Tregs. Table S1: Clinicopathological features of CD29 expression in LIHC. Table S2: List of primers used in this study. Table S3: List of antibodies used in this study. [file LIV-45-0-s001.zip › liv70421-sup-0003-FigureS2@Figure S2.png]

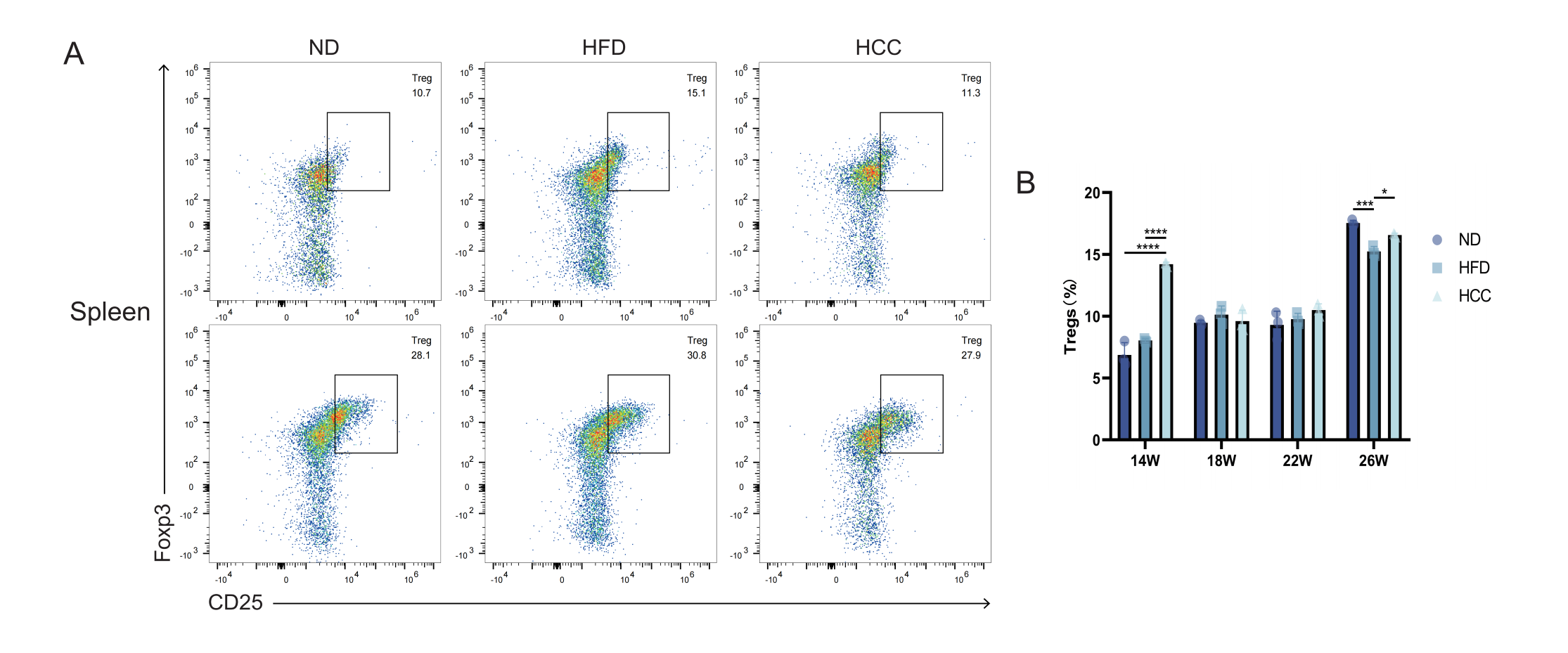

Supplement: Supplementary file 1 — Figure S1: Subpopulation composition of spleen T Cells in MASLD. Figure S2: Functional characteristics of T Cells in MASLD model. Figure S3: Subpopulation of CD8+ T Cells in MASLD. Figure S4: Composition and function of spleen Tregs in MASLD. Figure S5: Function characteristics of Tregs in MASLD model. Figure S6: Metabolic activity of Tregs and cell adhesion‐related genes in the pseudotime analysis trajectory of Tregs. Table S1: Clinicopathological features of CD29 expression in LIHC. Table S2: List of primers used in this study. Table S3: List of antibodies used in this study. [file LIV-45-0-s001.zip › liv70421-sup-0005-FigureS4@Figure S4.png]

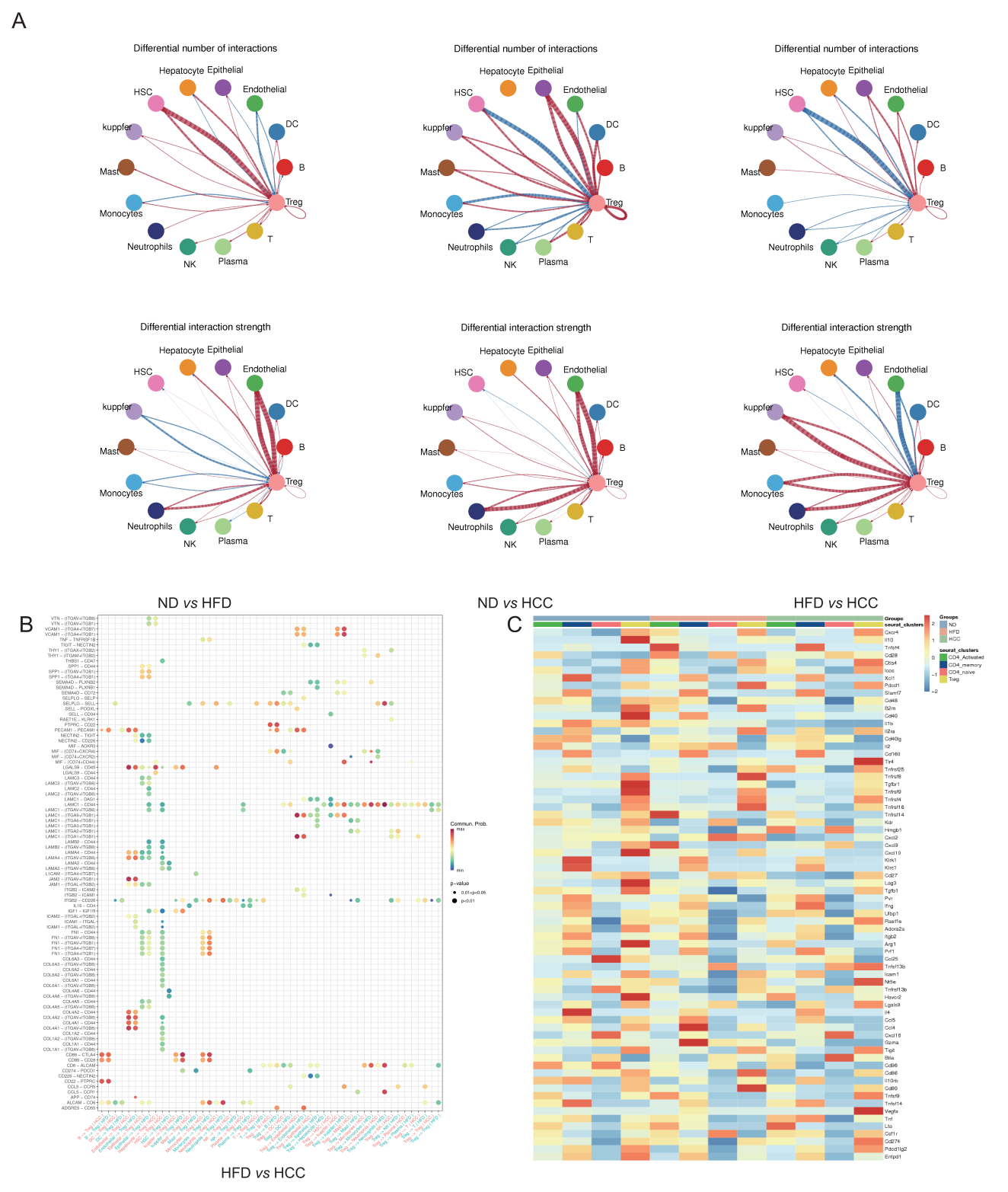

Supplement: Supplementary file 1 — Figure S1: Subpopulation composition of spleen T Cells in MASLD. Figure S2: Functional characteristics of T Cells in MASLD model. Figure S3: Subpopulation of CD8+ T Cells in MASLD. Figure S4: Composition and function of spleen Tregs in MASLD. Figure S5: Function characteristics of Tregs in MASLD model. Figure S6: Metabolic activity of Tregs and cell adhesion‐related genes in the pseudotime analysis trajectory of Tregs. Table S1: Clinicopathological features of CD29 expression in LIHC. Table S2: List of primers used in this study. Table S3: List of antibodies used in this study. [file LIV-45-0-s001.zip › liv70421-sup-0006-FigureS5@Figure S5.png]

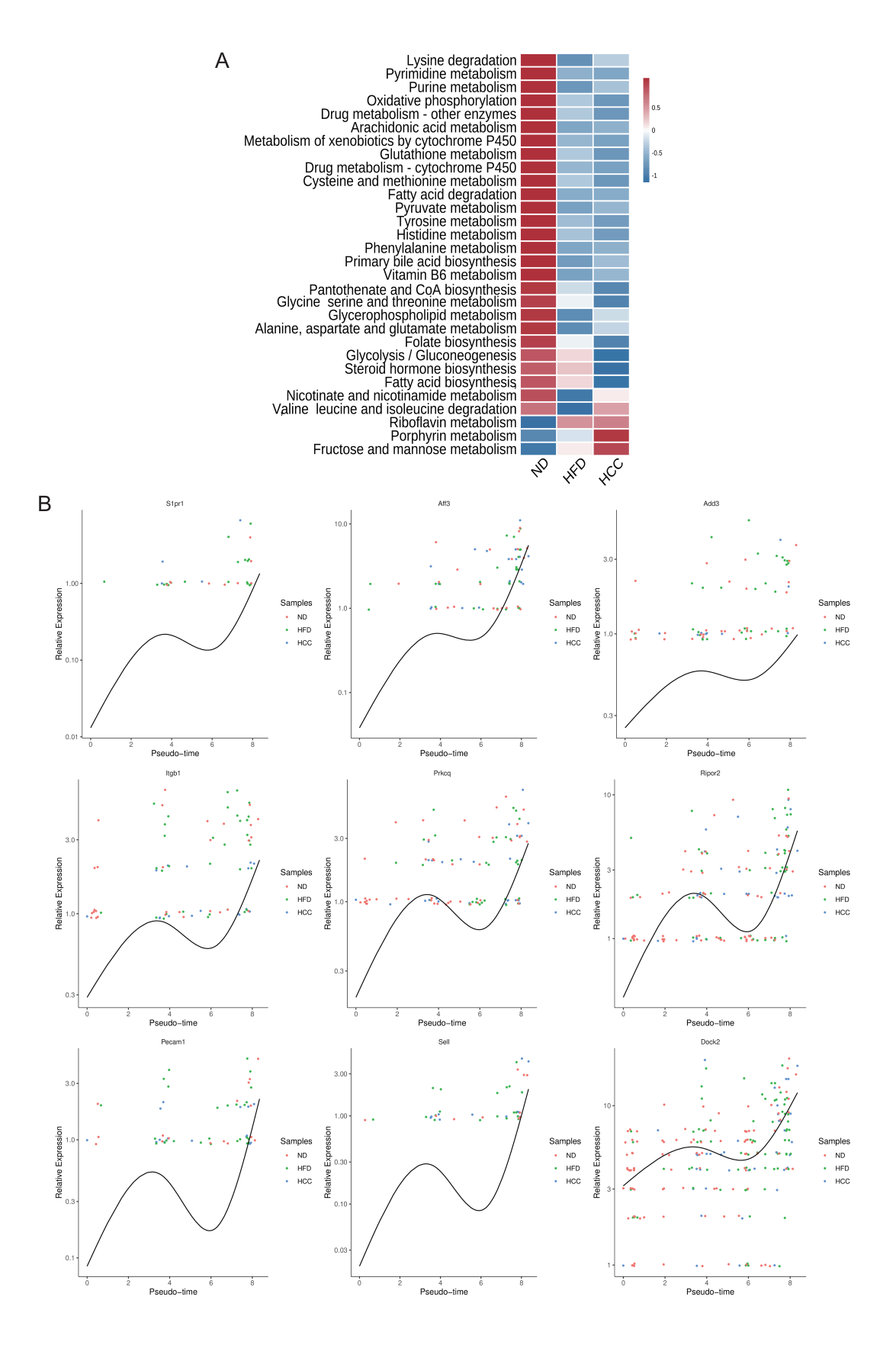

Supplement: Supplementary file 1 — Figure S1: Subpopulation composition of spleen T Cells in MASLD. Figure S2: Functional characteristics of T Cells in MASLD model. Figure S3: Subpopulation of CD8+ T Cells in MASLD. Figure S4: Composition and function of spleen Tregs in MASLD. Figure S5: Function characteristics of Tregs in MASLD model. Figure S6: Metabolic activity of Tregs and cell adhesion‐related genes in the pseudotime analysis trajectory of Tregs. Table S1: Clinicopathological features of CD29 expression in LIHC. Table S2: List of primers used in this study. Table S3: List of antibodies used in this study. [file LIV-45-0-s001.zip › liv70421-sup-0007-FigureS6@Figure S6.png]
